# Supplementary material for: Ethane-oxidising archaea couple CO2 generation to F420 reduction
Source: Nat Commun. 2024 Oct 21;15:9065. doi: 10.1038/s41467-024-53338-7 (PMC11493965; doi:10.1038/s41467-024-53338-7)
Supplement: Supplementary file 1 — Supplementary Information [file 41467_2024_53338_MOESM1_ESM.pdf]

# Supplementary Materials

## Ethane-oxidising archaea couple $CO_2$ generation to $F_{420}$ reduction

Olivier N Lemaire, Gunter Wegener, and Tristan Wagner

Correspondence to: twagner@mpi-bremen.de

**This PDF file includes:**

Supplementary Figures 1 to 19
Supplementary Tables 1 to 5

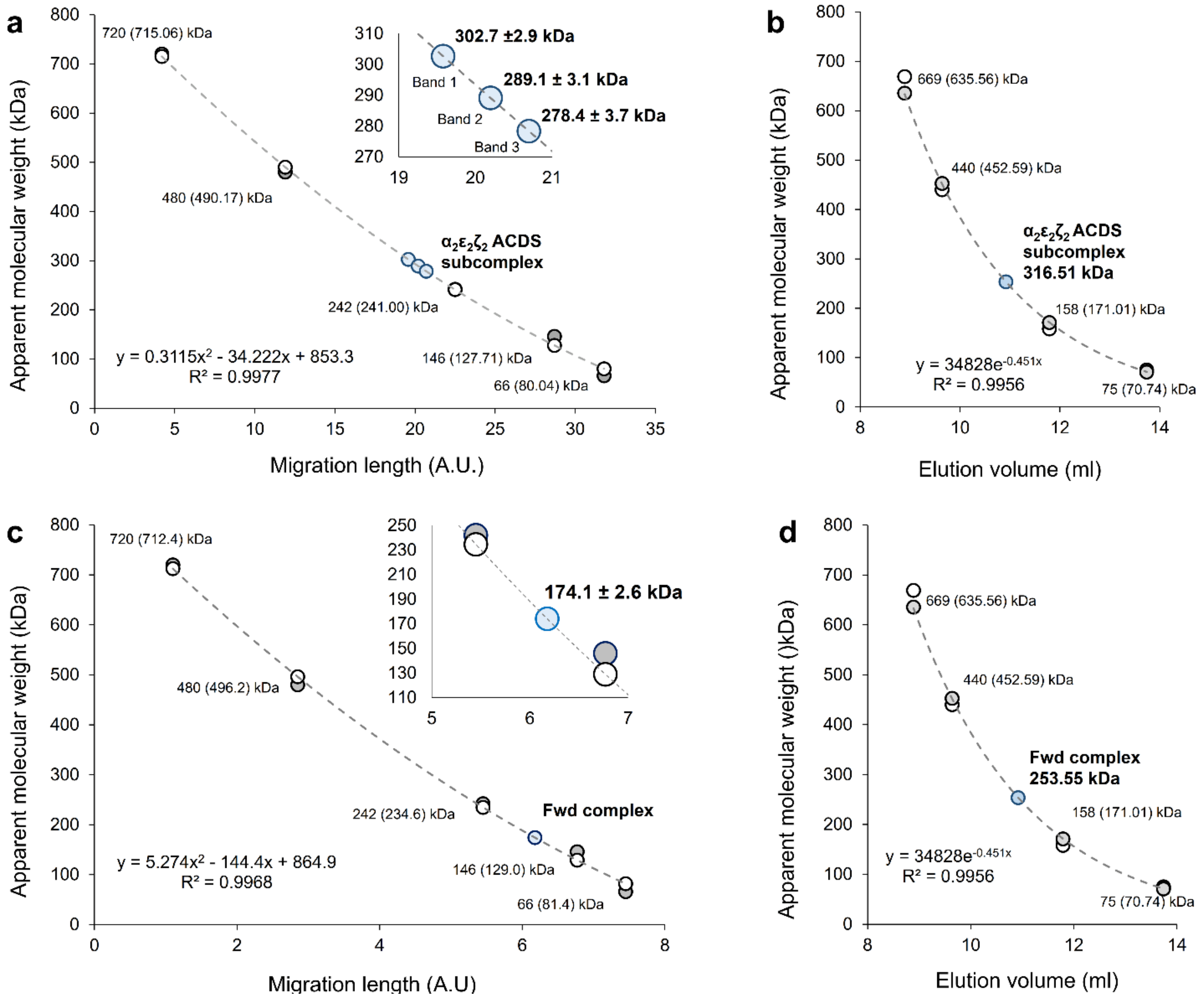


**Supplementary Fig. 1. Size estimation of the complexes purified from *Ca.* E. thermophilum on native PAGE and size exclusion chromatography. a and c**, Size estimation of the ACDS CODH subcomplex (**a**, theoretical molecular weight of 294,308 Da for $\alpha_2\varepsilon_2\zeta_2$) and Fwd complex (**c**, theoretical molecular weight of 199,467 Da for FwdABCDGI) on native gels. The size and migration length of the proteins from the commercial ladder (prestained PageRuler from ThermoScientific, Germany) were used to calculate a fit (dashed grey line, equation, and $R^2$ indicated) to determine the molecular weights. White and grey dots correspond to the theoretical and measured molecular weights from the protein ladder. Blue dots display the molecular weight calculated from the fit for ACDS CODH subcomplex and Fwd enzymes. The insert shows a close-up of the graph to describe the size of the different bands stained by CODH activity. **b** and **d**, Size determination of the ACDS CODH subcomplex (**b**) and Fwd complex (**d**) by size-exclusion chromatography using elution volumes of standard proteins (High Molecular Weight range Gel Filtration Calibration Kit, GE Healthcare, Munich, Germany) and the purified enzymes. The size and elution volumes of the protein from the commercial ladder (white dots) were used to calculate a fit (dashed grey line, equation, and $R^2$ indicated), used for the determination of the theoretical size of the standard proteins (grey dots) and the proteins from *Ca.* E. thermophilum (blue dots). Source data are provided as a Source Data file.

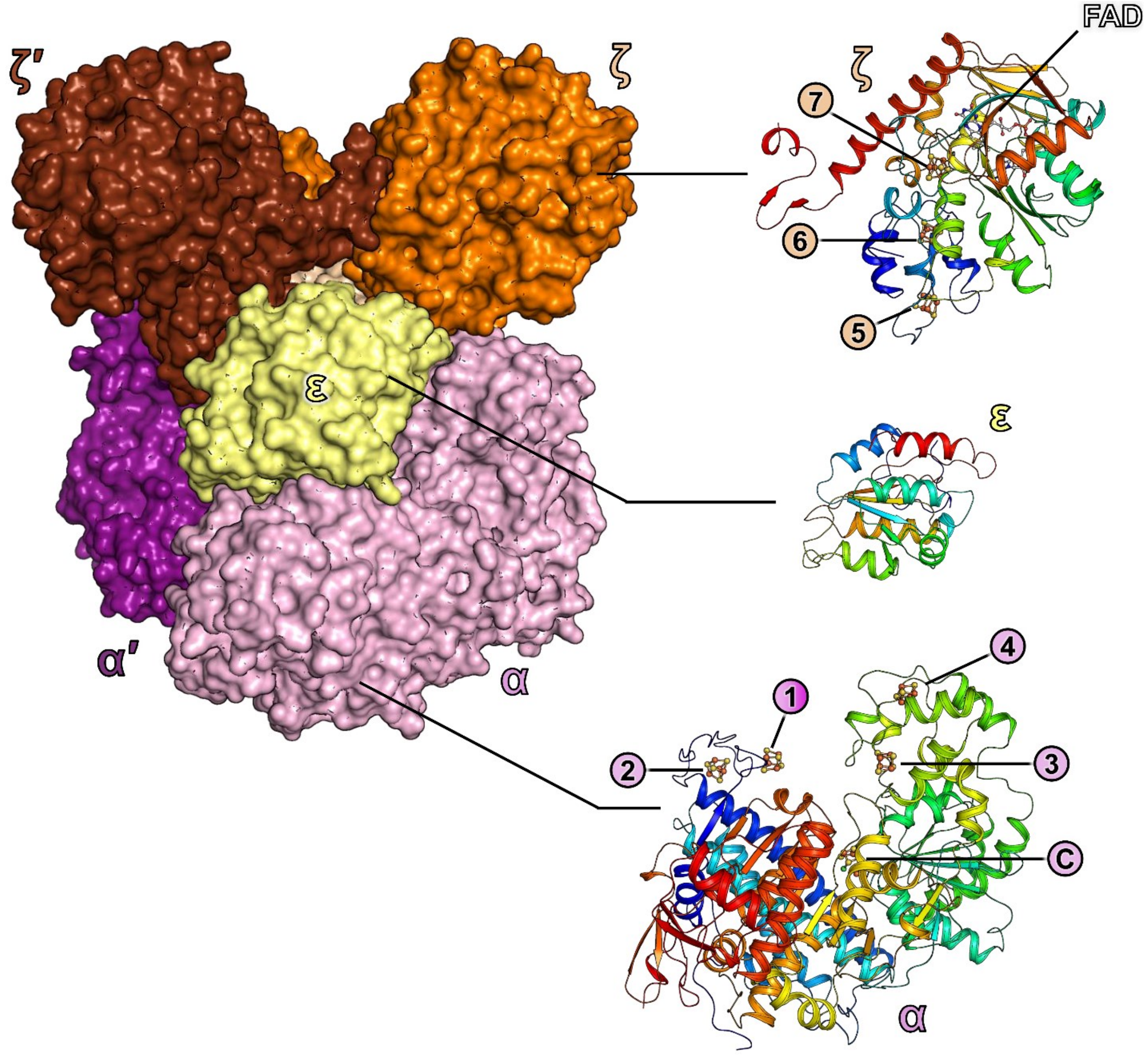


**Supplementary Fig. 2. Structure of the individual components of the $\alpha_2\varepsilon_2\zeta_2$ subcomplex from *Ca*. E. thermophilum.** The complex is shown in the upper left panel as a surface with α, ε, ζ, α′, ε′, and ζ′ subunits coloured light pink, light yellow, orange, deep purple, wheat, and brown, respectively. Individual α, ε, and ζ subunits are presented in cartoons and coloured in a rainbow from blue to red (N-terminus to C-terminus) in the same pose as the top left panel. Cofactors are displayed as balls and sticks and atoms coloured white, red, blue, yellow, light orange, and orange for carbon, oxygen, nitrogen, sulphur, phosphorus, and iron, respectively.

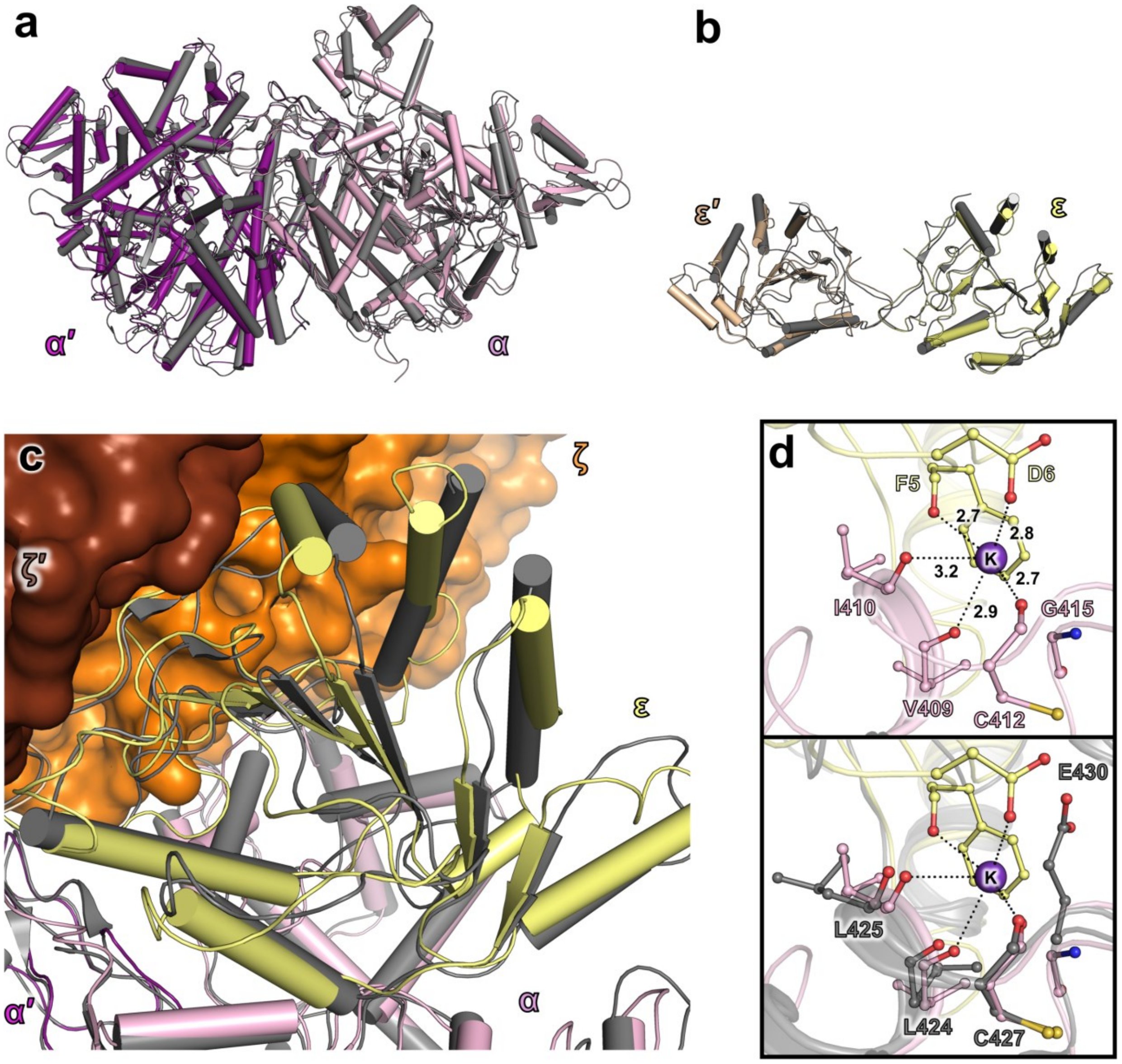


**Supplementary Fig. 3. Alignment of $\alpha_2\varepsilon_2\zeta_2$ subcomplex from *Ca*. E. thermophilum and the $\alpha_2\varepsilon_2$ subcomplex from *M. barkeri.*** **a-b**, Structural alignment of the ACDS $\alpha_2\varepsilon_2\zeta_2$ subcomplex and its equivalent from *M. barkeri* (PDB 3CF4[1]). **a**, Structural alignment of the αα′ core from the *M. barkeri* (grey cartoon) on the αα′ core of the subcomplex from *Ca*. E. thermophilum (light pink and deep purple, respectively). The r.m.s.d. and coverage are given in Supplementary Table 4. **b**, Superimposition of the structure of the ε subunit from the *M. barkeri* (grey cartoon) on both the ε and ε′ subunits of the subcomplex from *Ca*. E. thermophilum (light yellow and wheat cartoon, respectively). **c**, The $\alpha_2\varepsilon_2$ subcomplex from *Ca*. E. thermophilum and *M. barkeri* are shown as cartoons, coloured as in **a-b**, and aligned on the αα′ core. The ζζ′ dimer is shown in surface, with subunits coloured orange and brown, respectively. **d**, Top: Coordination of the ion modelled as a K atom (purple sphere) in the structure from *Ca.* E. thermophilum at the interface of the α (pink) and ε (light yellow) subunits. Bottom: superimposition with the structure of the subcomplex from *M. barkeri* (grey). Proteins are shown as cartoons, and the residues in the vicinity are shown as balls and sticks with oxygen, nitrogen and sulphur coloured red, blue and yellow, respectively, with contacts shown as black dashes. Labelled distances are in Å. C412 coordinates the cluster 3.

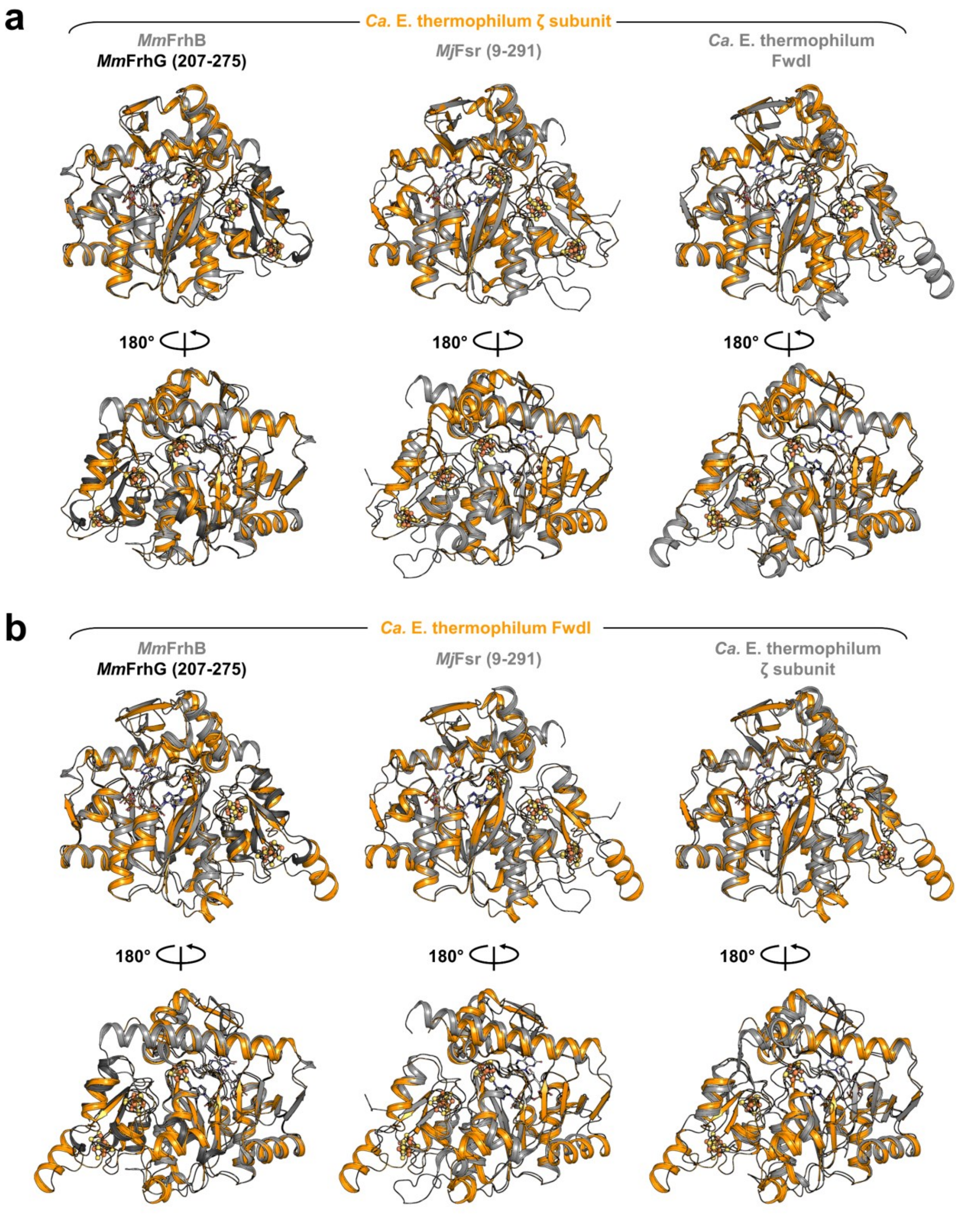


**Supplementary Fig. 4. Structural alignments of the ζ subunit from the CODH component of the ACDS and FwdI subunit from the Fwd complex of *Ca*. E. thermophilum with structurally characterised homologues. a-b**, Alignment of the ζ subunit (**a**) or the FwdI subunit (**b**) with the structures of the FrhBG (γ207-275) subcomplex from *M. marburgensis* (PDB 4OMF) and Fsr (9-291) from *Methanocaldococcus jannaschii* (PDB 7NP8). The alignment r.m.s.d. and coverage are given in Supplementary Table 4.

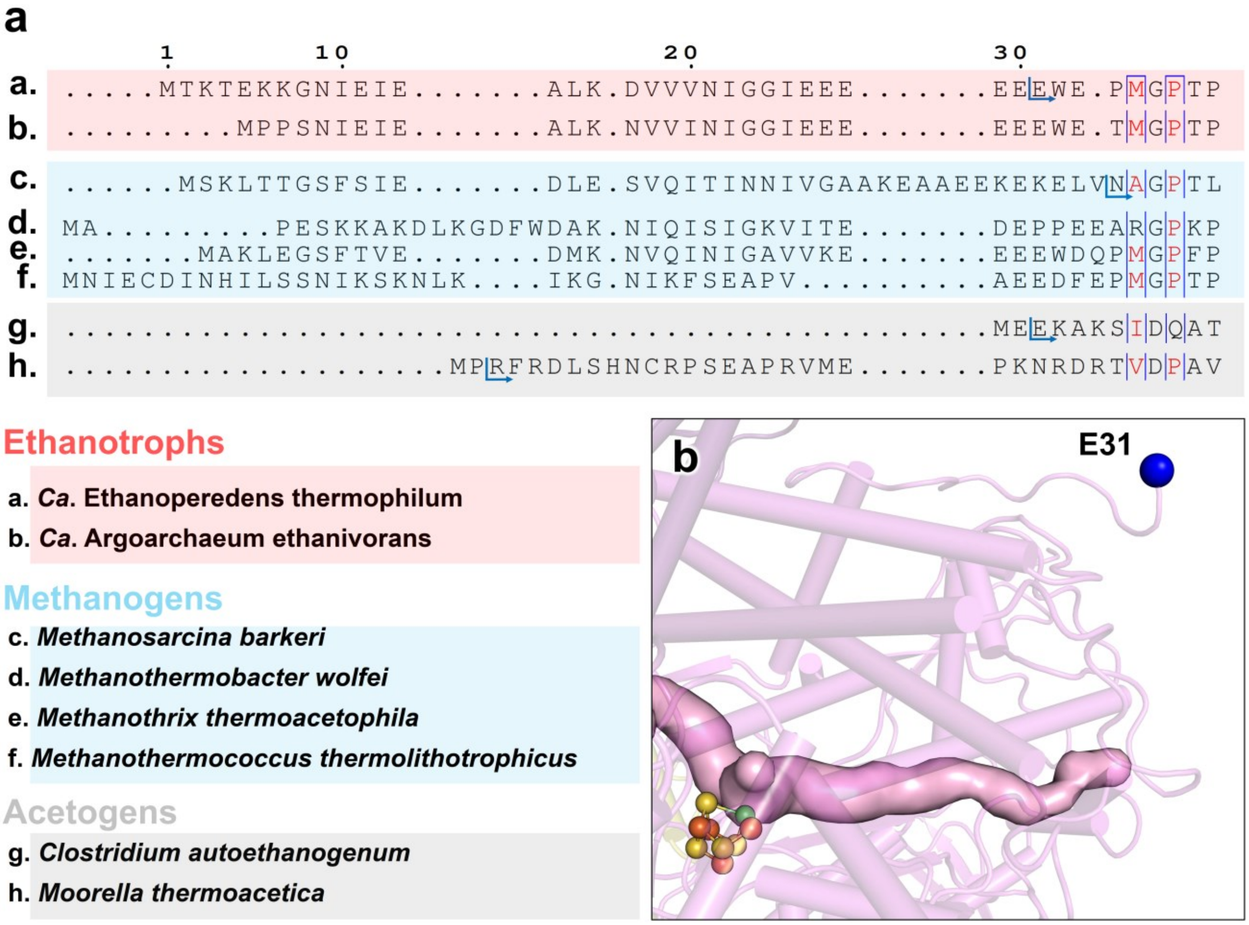


**Supplementary Fig. 5. N-terminal extension in CO-dehydrogenases. a**, Multiple alignments of the sequences of CODH (ACDS α-subunit) in archaea and bacteria. The numbering corresponds to the sequence of the protein from *Ca*. E. thermophilum. A blue arrow indicates the beginning of the chain in the respective structural model[1-3]. **b**, Position of the first modelled residue of the α-subunit from *Ca*. E. thermophilum. The protein is shown as a cartoon, coloured light pink, with the C-cluster shown as balls and sticks with sulphur, nickel, and iron coloured yellow, green, and orange, respectively. The N-terminus of the model is represented as a blue ball. The CO-diffusion channel predicted by the CAVER program is shown as a light pink surface.

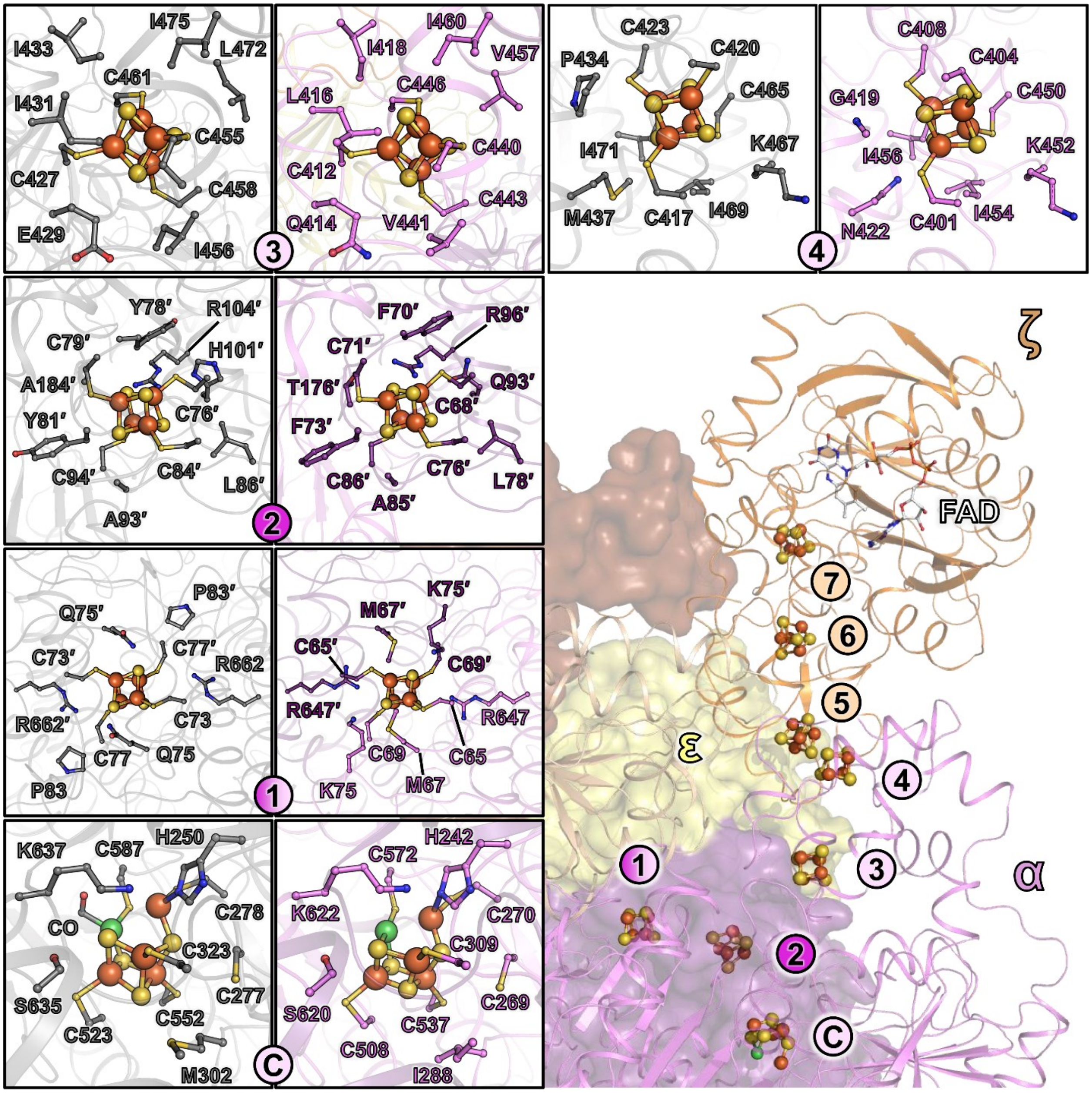


**Supplementary Fig. 6. Metallo-cofactors coordination in the $\alpha_2\varepsilon_2$ subcomplex from *Ca*. E. thermophilum.** The α, ε and ζ subunits are represented as cartoons and the α′, ε′ and ζ′ subunits as a surface to provide an overall view of the metallo-cofactor locations. Framed panels display a close-up view of the coordination comparing the $\alpha_2\varepsilon_2$-subcomplex from *M. barkeri* (PDB 3CF4, grey) and *Ca*. E. thermophilum (coloured as in the overall view). The different cofactors and residues in their vicinity are represented in balls and sticks with nitrogen, oxygen, sulphur, phosphorus, nickel, and iron coloured in blue, red, yellow, light orange, green, and orange. Carbon atoms of the FAD cofactor are coloured white. The coordination of the cofactors from the ζ subunit is described in Supplementary Figs. 8 and 9.

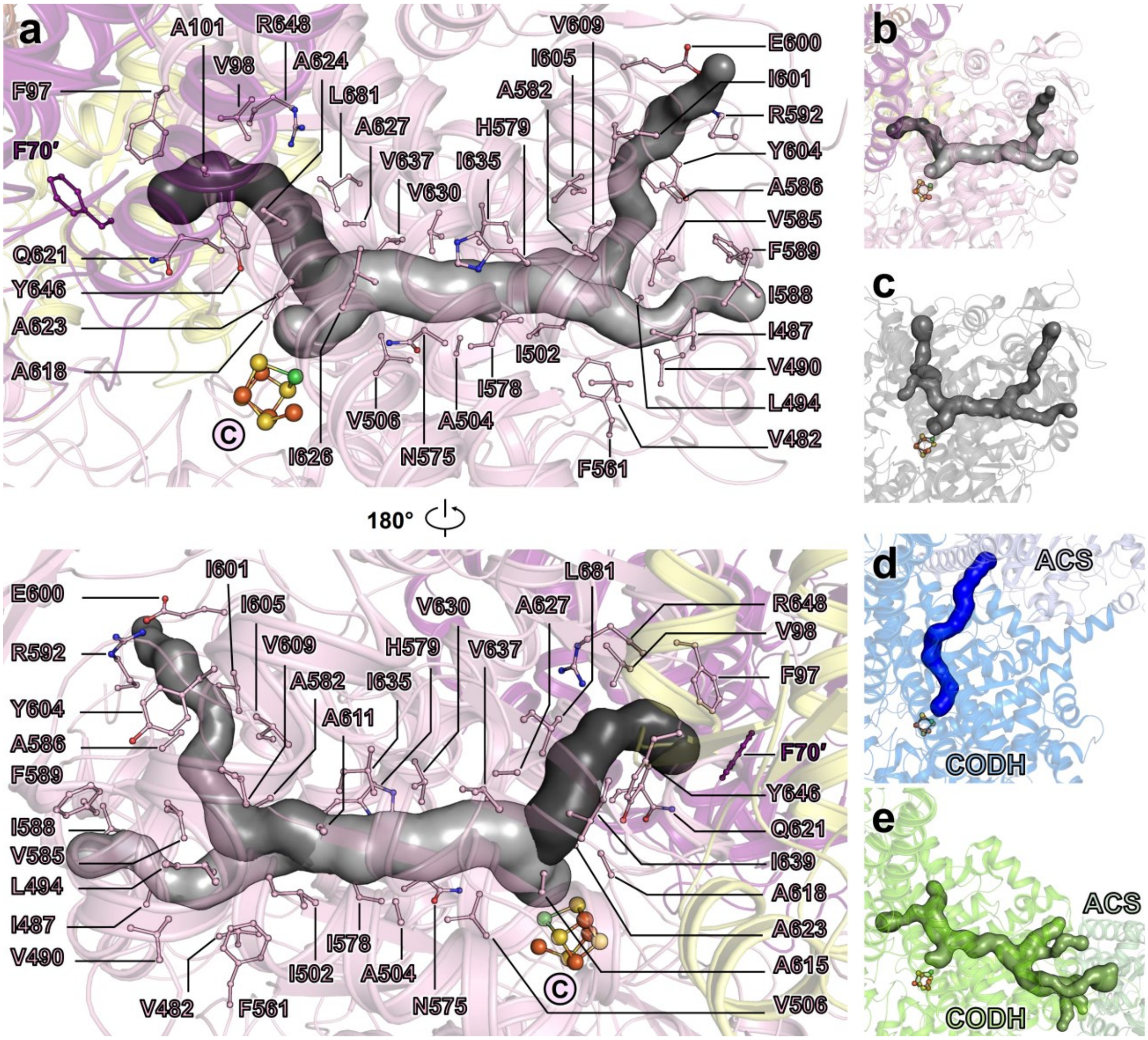


**Supplementary Fig. 7. Tunnelling systems of the $\alpha_2\varepsilon_2\zeta_2$ ACDS from *Ca*. E. thermophilum. a**, The different tunnels predicted by the CAVER program (interrupted at the hydrophilic bottleneck near F70′) are shown as surface and coloured in shades of grey. The proteins are shown in cartoons with α, α′ and ε subunits coloured light pink, deep purple, and light yellow, respectively. The residues forming the channels and cofactors are shown in balls and sticks with oxygen, nitrogen, sulphur, iron, and nickel atoms coloured in red, blue, yellow, orange, and green, respectively. **b-e**, Tunnelling systems comparison between the $\alpha_2\varepsilon_2\zeta_2$ ACDS from *Ca.* E. thermophilum (**b**), the $\alpha_2\varepsilon_2$ ACDS subcomplex from *M. barkeri* (grey, PDB 3CF4, **c**), the CODH/acetyl-coenzyme A synthase (ACS) from *Moorella thermoacetica* (PDB 1MJG, **d**), and the CODH/ACS from *Clostridium autoethanogenum* (PDB 6YTT, **e**). The C-cluster is shown as balls and sticks with sulphur, iron, and nickel coloured yellow, orange, and green, respectively. **d and e**, the structures are coloured in shades of blue and green, respectively, with the CODH in lighter and ACS in darker colours.

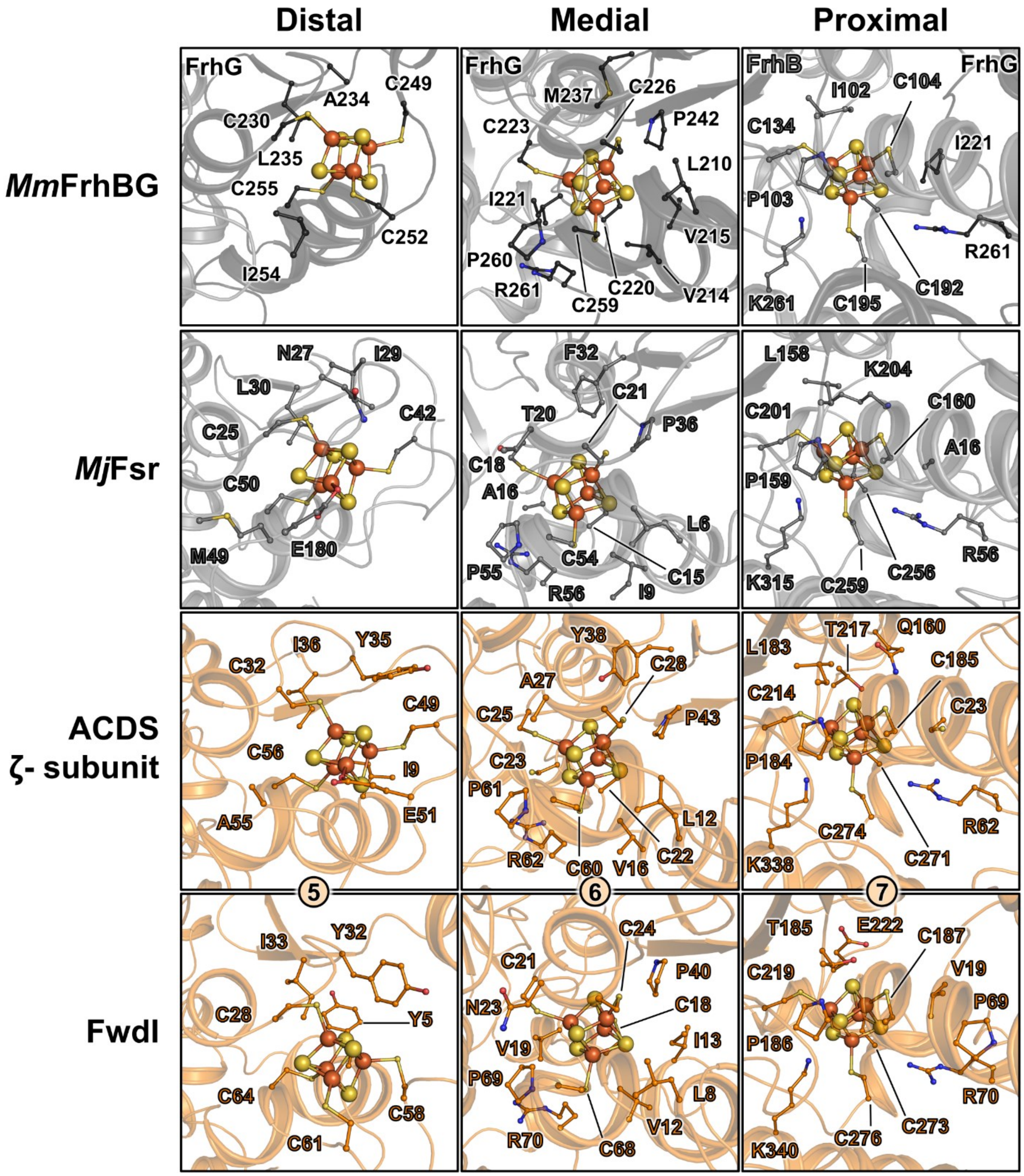


**Supplementary Fig. 8. Metallo-cofactor coordination in the $F_{420}$ reductases from *Ca*. E. thermophilum and related structures.** The coordination of the distal, medial and proximal clusters in the *Mm*FrhBG structure (PDB 4OMF, with distal and medial clusters belonging to FrhG subunit in black and the proximal cluster belonging to FrhB in grey, 1st raw), *Mj*Fsr (PDB 7NP8, coloured in grey, 2nd raw), the ACDS ζ subunit (coloured in orange, 3rd raw) and the FwdI subunit of the Fwd complex (coloured in orange, 4th raw) is shown. The proteins are displayed as cartoons, with the clusters and the surrounding residues shown as balls and sticks with oxygen, nitrogen, sulphur, and iron coloured in red, blue, yellow, and orange, respectively.

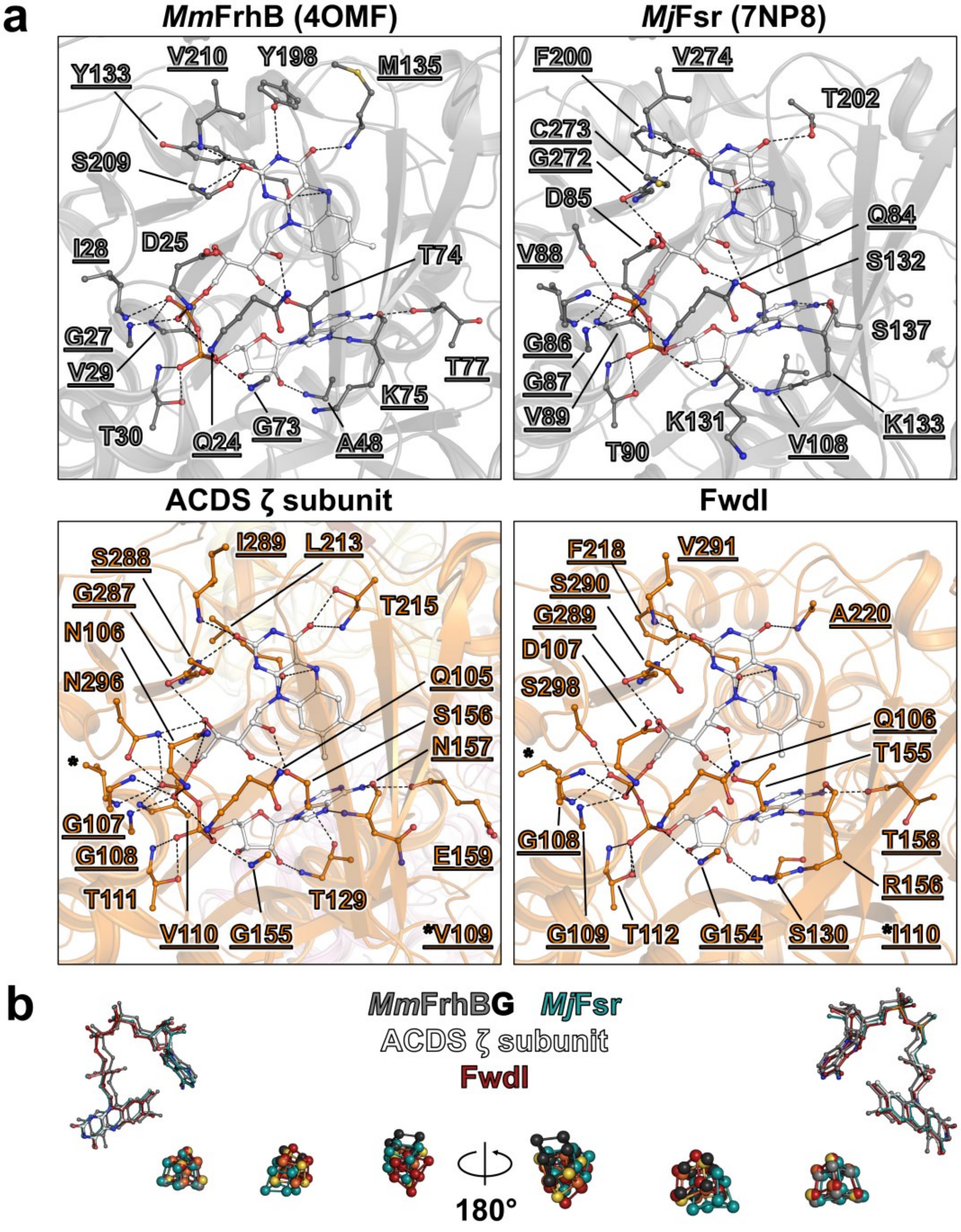


**Supplementary Fig. 9. FAD coordination in the $F_{420}$ reductases from *Ca*. E. thermophilum and related structures. a**, Proteins are displayed in cartoons, coloured grey (*Mm*FrhB and *Mj*Fsr) or orange (ACDS ζ subunit and FwdI). FAD is coloured white. The residues surrounding the FAD are shown as balls and sticks, with contacts shown as black dashes. Residues in contact with the FAD by their main or side chain are labelled underlined or not, respectively (and not underlined if both). Oxygen, nitrogen, sulphur, and phosphorus are coloured red, blue, yellow and light orange, respectively. The carbons from the FAD are coloured white. **b**, Superimposition of the (metallo)-cofactors of the different structures, coloured as indicated. The structural alignment was done using the complete ACDS ζ subunit, the complete FwdI subunit, the FrhBG (γ207-275) subcomplex (PDB 4OMF) and Fsr (9-291, PDB 7NP8). In the ACDS ζ subunit, atoms are coloured per type, as in **a**.

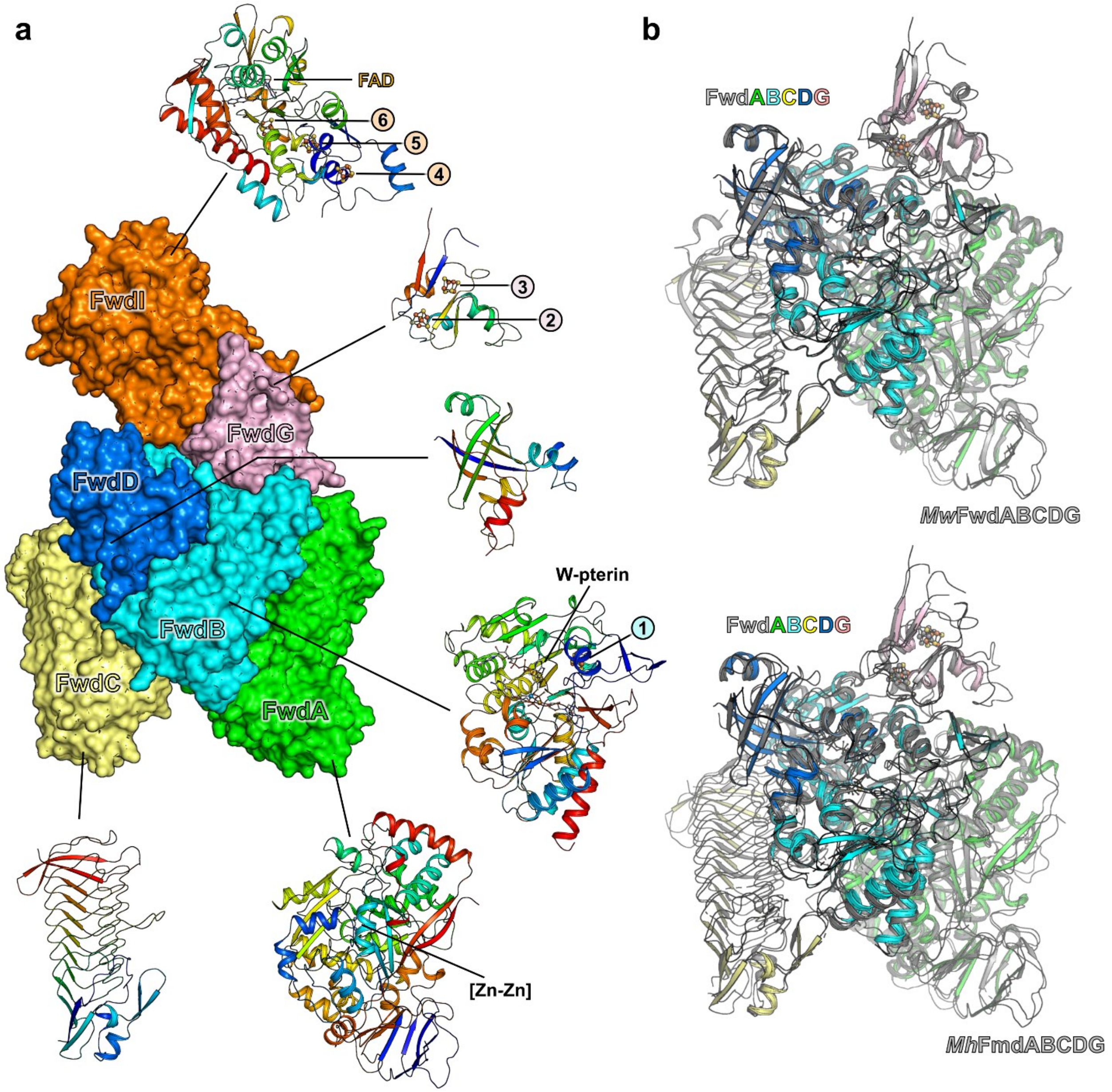


**Supplementary Fig. 10. Structure of the individual components of the Fwd complex from *Ca*. E. thermophilum and superposition with structural homologues. a**, Structure of the Fwd complex (surface) and the individual subunits (cartoon). The individual subunits are coloured from the N-terminus to the C-terminus in a rainbow gradient from blue to red, in the same pose as the top left panel. Cofactors are shown as balls and sticks with carbon, oxygen, nitrogen, sulphur, phosphorus, iron, zinc and tungsten coloured white, red, blue, yellow, light orange, orange, grey and blue-grey, respectively. **b**, Structural alignment of the Fwd complex from *Ca*. E. thermophilum (coloured as in **a**), the FwdABCDG core of the Fwd complex from *M. wolfei* (PDB 5T5M, coloured in grey, top), and the FmdABCDG core of the Fmd-containing supercomplex from *M. hungatei* (PDB 7BKB, coloured in grey, bottom). The proteins are shown as cartoons with cofactors displayed as balls and sticks and coloured as in **a**. The alignment r.m.s.d. and coverage are given in Supplementary Table 4.

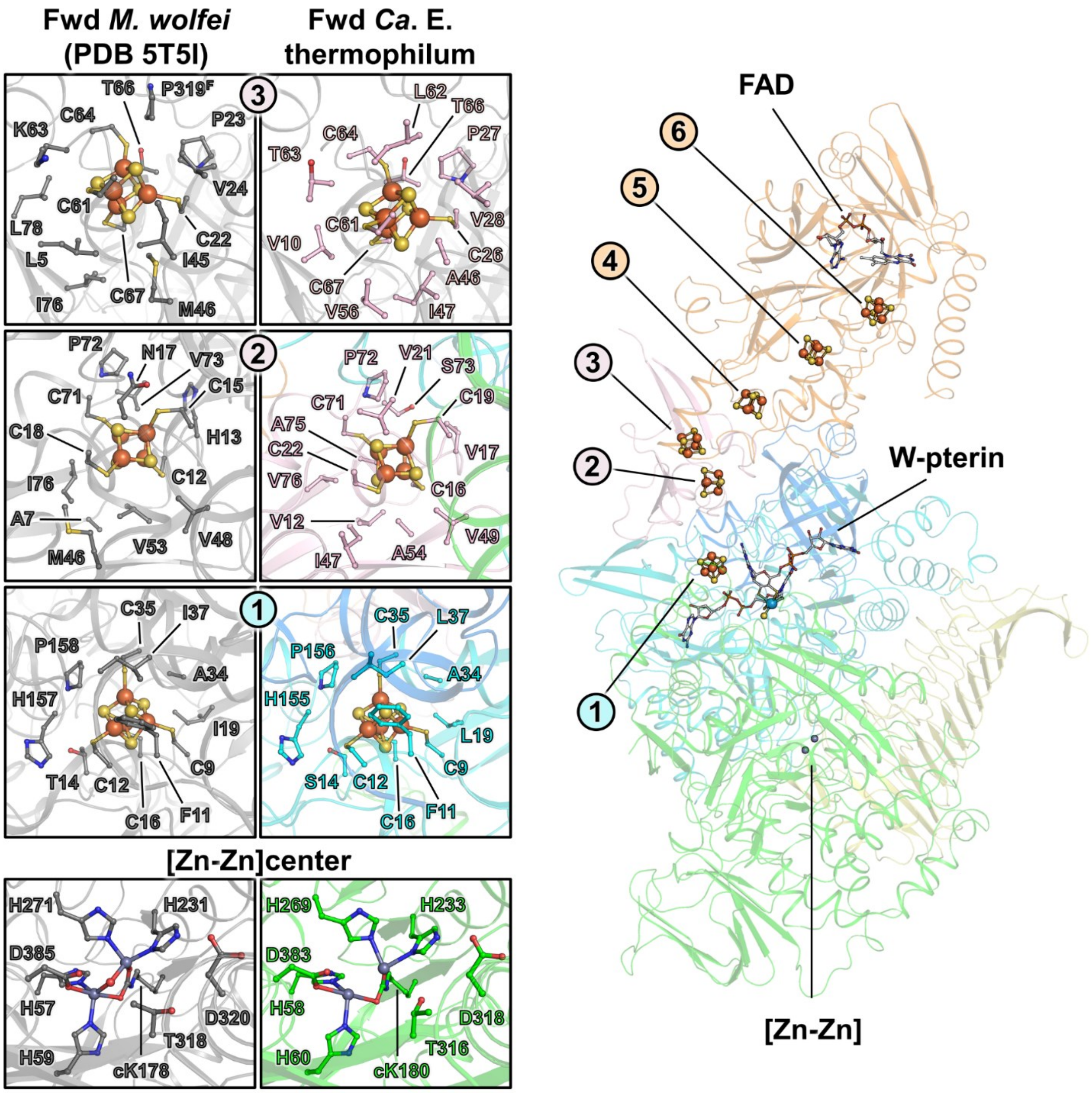


**Supplementary Fig. 11. Cofactors coordination in the Fwd complex from *Ca*. E. thermophilum and *M. wolfei*.** The proteins are represented as cartoons with the A, B, C, D, G, and I subunits of the Fwd complex from *Ca*. E. thermophilum coloured green, cyan, light yellow, marine blue, light pink, and orange, respectively. The proteins from *M. wolfei* are coloured grey. The different cofactors and residues in their vicinity are represented as balls and sticks with nitrogen, oxygen, sulphur, phosphorus, zinc, tungsten, and iron coloured in blue, red, yellow, light orange, grey, grey-blue, and orange. Carbon atoms of the FAD and tungstopterin cofactors are coloured white. The P319$^{F}$ is labelled $^{F}$ because the residue is part of the FwdF subunit instead of FwdG.

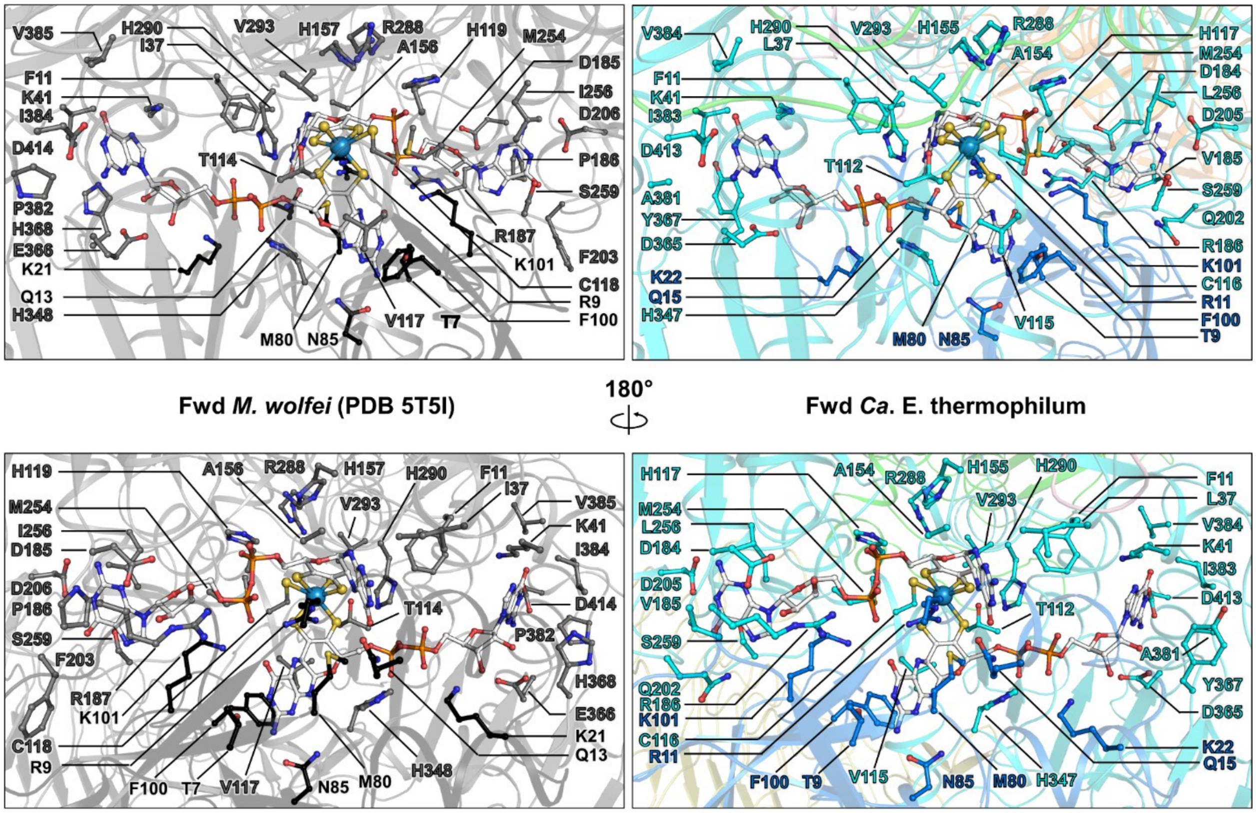


**Supplementary Fig. 12. Tungstopterin coordination in the Fwd complex from *Ca*. E. thermophilum and *M. wolfei*.** The proteins are shown as cartoons with the A, B, C, D, and G subunits of the Fwd complex from *Ca*. E. thermophilum coloured green, cyan, light yellow, marine blue, and light pink, respectively. All subunits from *M. wolfei* are coloured grey except for FwdD, which is coloured black. The tungstopterin and residues in its vicinity are represented in balls and sticks with nitrogen, oxygen, sulphur, phosphorus, and tungsten coloured blue, red, yellow, light orange, and grey blue. Carbon atoms of the tungstopterin are coloured white.

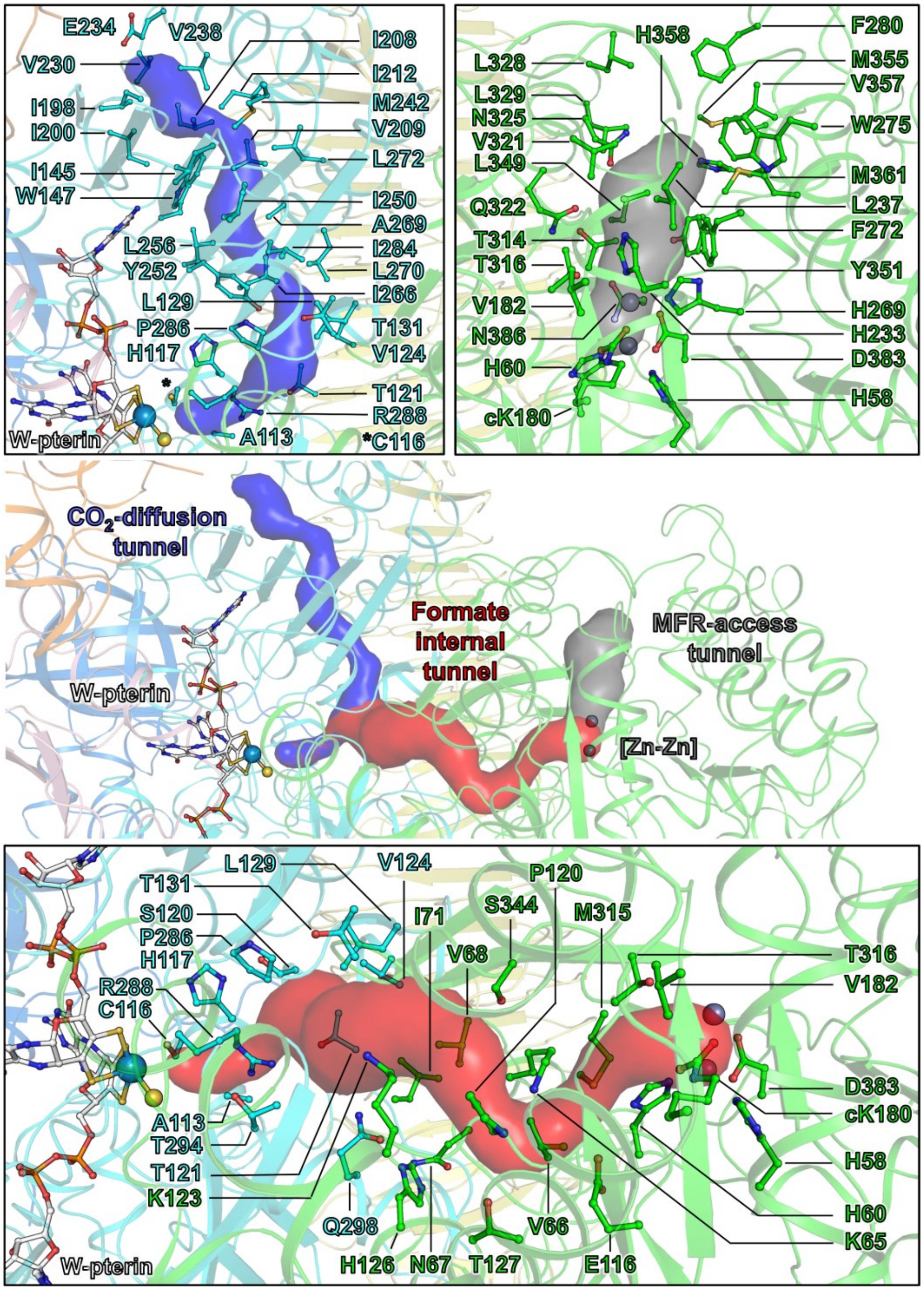


**Supplementary Fig. 13. Tunnelling systems of the Fwd complex from *Ca*. E. thermophilum.** The central panel presents the overall structure and the different functional tunnels predicted by the CAVER program (displayed as transparent surfaces). Details of the tunnel composition are shown in the framed subpanels. The subunits are represented as cartoons with the A, B, C, D, G, and I subunits coloured green, cyan, light yellow, marine blue, light pink, and orange, respectively. The residues structuring each tunnel and metallo-cofactors are displayed as balls and sticks with oxygen, nitrogen, sulphur, phosphorus, zinc, and tungsten atoms coloured red, blue, yellow, light orange, dark grey, and blue-grey, respectively. Tungstopterin carbons are coloured white.

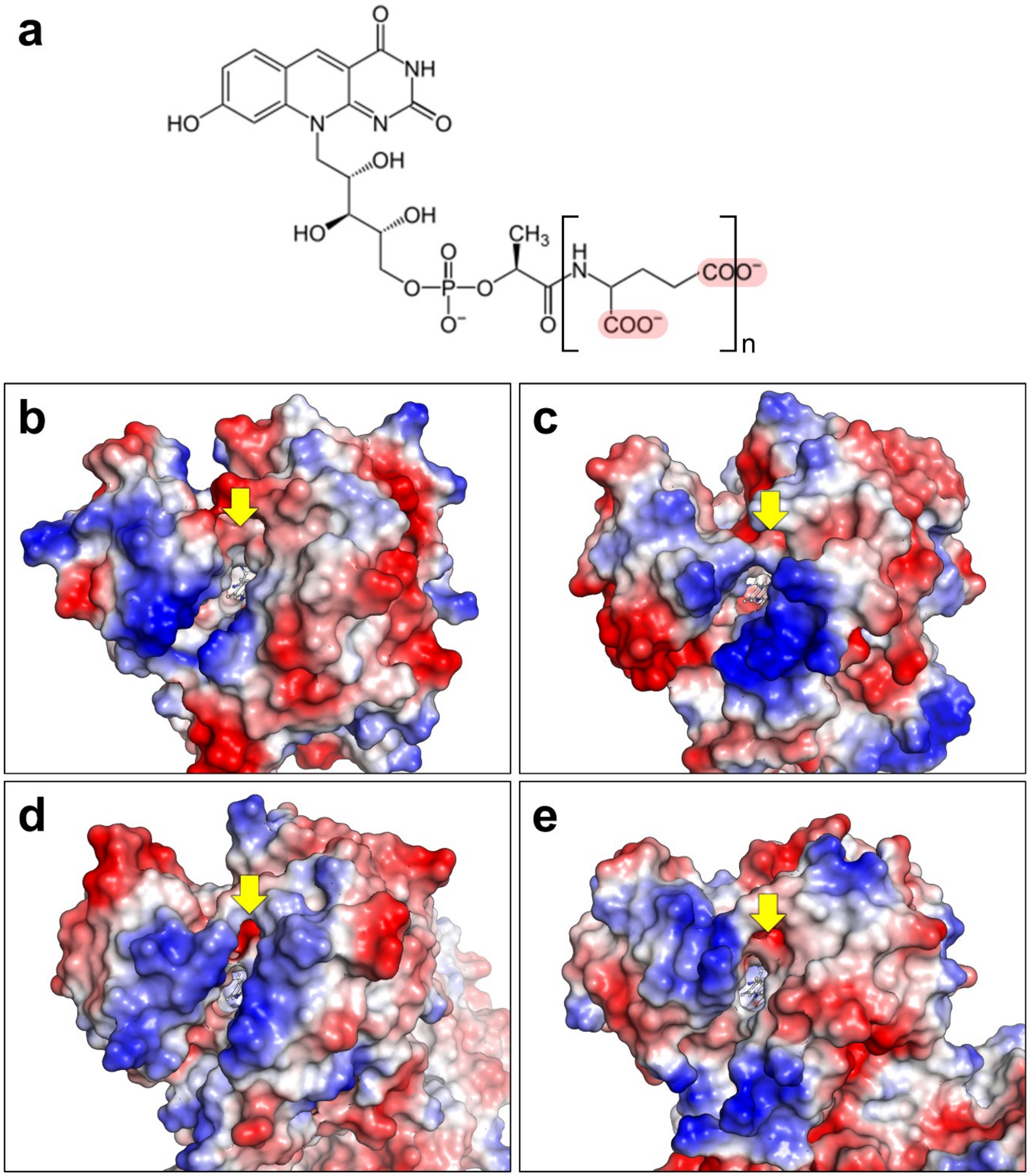


**Supplementary Fig. 14. Charge distribution and $F_{420}$ stabilisation around the FAD binding site. a**. Structure of the $F_{420}$ molecule in methanogenic archaea. The negative charges harboured on the polyglutamate tail are highlighted in red. **b-e**. Charge distribution around the $F_{420}$ binding site of the ACDS ζ subunit (**b**), FwdI (**c**), *Mm*FrhB (**d**, 4OMF) and *Mj*Fsr (**e**, 7NP8). Protein surfaces are coloured from blue to red, corresponding to positive and negative charges, respectively. Buried FAD cofactors are displayed in balls and sticks with carbon, oxygen, and nitrogen, coloured white, red, and blue, respectively. A yellow arrow points to the cleft expected to host the isoalloxazine $F_{420}$ group. The positively charged regions around the $F_{420}$ binding site are supposed to stabilise the negative glutamate groups of the coenzyme.

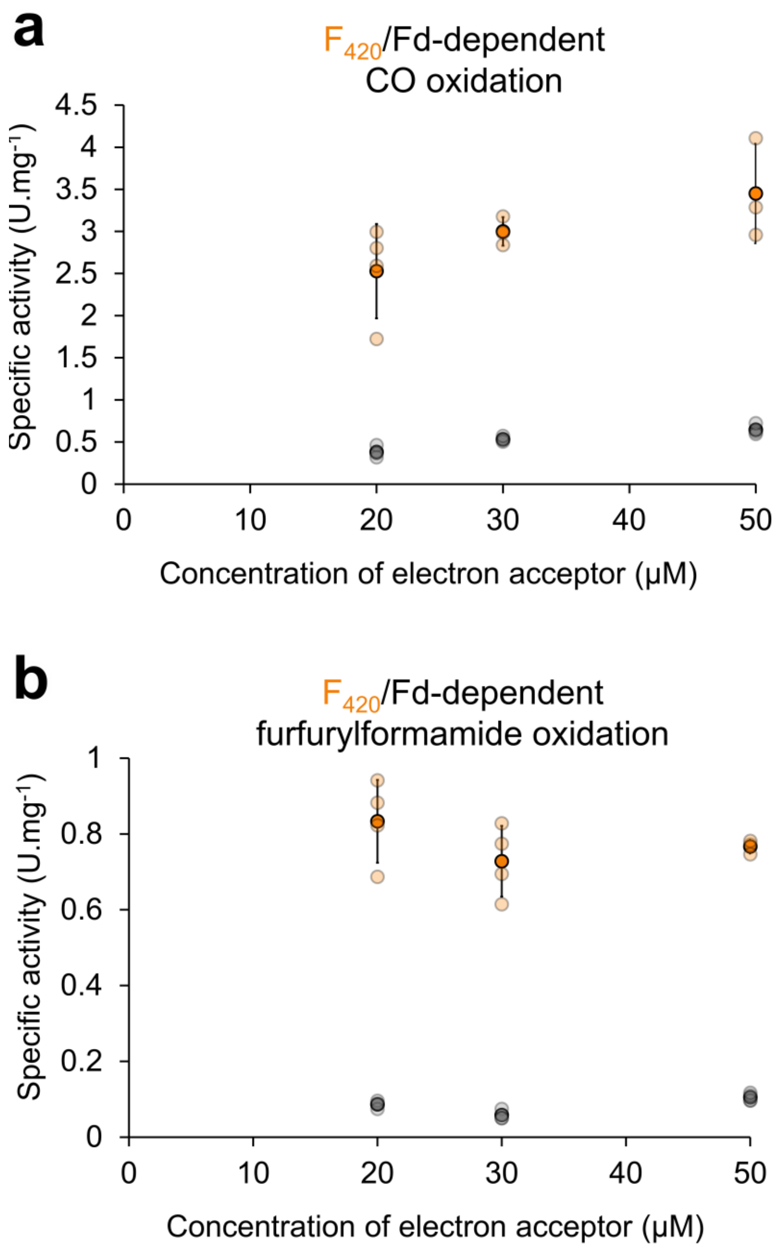


**Supplementary Fig. 15. Enzymatic activity of the purified complexes with $F_{420}$ and ferredoxin as electron acceptors.** The CO oxidation catalysed by the CODH component of the ACDS (**a**) and the furfurylformamide oxidation catalysed by the Fwd complex (**b**) were monitored in the presence of different concentrations of $F_{420}$ (orange circles) or the ferredoxin (grey circles) from *C. autoethanogenum*[4] as electron acceptor. Units correspond to µmol of substrate oxidised, assuming that one and two electrons are carried by the ferredoxin and $F_{420}$, respectively. In comparison, the measured Fd-dependent CO oxidase-specific activity from the CODH of *C. autoethanogenum*[4] is 137 $U.min.^{-1}.mg^{-1}$. Average and standard deviations are plotted (n= 3 or 4 independent measurements, see Source Data file), and the individual data are shown as transparent circles. The activity of the enzyme with 5 mM MV as acceptor was 5.63 ± 0.94 (CODH component) and 2.15 ± 0.26 (Fwd) µmol of oxidised substrate per min per mg of enzymes, respectively. Source data are provided as a Source Data file.

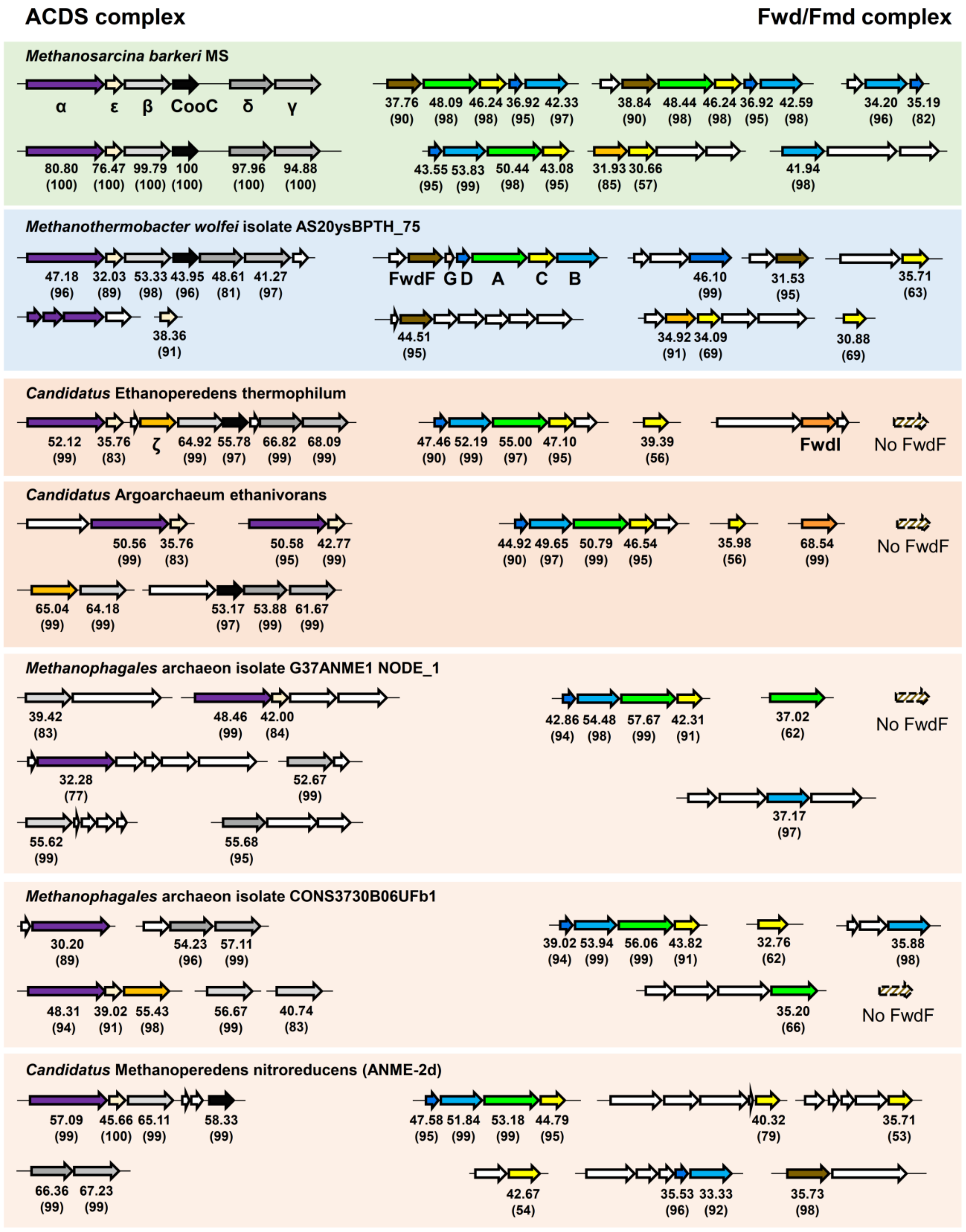

ACDS complex
Fwd/Fmd complex
Methanosarcina barkeri MS
α ε β CooC δ γ
37.76 48.09 46.24 36.92 42.33
(90) (98) (98) (95) (97)
38.84 48.44 46.24 36.92 42.59
(90) (98) (98) (95) (98)
34.20 35.19
(96) (82)
80.80 76.47 99.79 100 97.96 94.88
(100) (100) (100) (100) (100) (100)
43.55 53.83 50.44 43.08
(95) (99) (98) (95)
31.93 30.66
(85) (57)
41.94
(98)
Methanothermobacter wolfei isolate AS20ysBPTH_75
47.18 32.03 53.33 43.95 48.61 41.27
(96) (89) (98) (96) (81) (97)
FwdF G D A C B
46.10
(99)
31.53
(95)
35.71
(63)
38.36
(91)
44.51
(95)
34.92 34.09
(91) (69)
30.88
(69)
Candidatus Ethanoperedens thermophilum
52.12 35.76 ζ 64.92 55.78 66.82 68.09
(99) (83) (99) (97) (99) (99)
47.46 52.19 55.00 47.10
(90) (99) (97) (95)
39.39
(56)
FwdI
No FwdF
Candidatus Argoarchaeum ethanivorans
50.56 35.76
(99) (83)
50.58 42.77
(95) (99)
44.92 49.65 50.79 46.54
(90) (97) (99) (95)
35.98
(56)
68.54
(99)
No FwdF
65.04 64.18
(99) (99)
53.17 53.88 61.67
(97) (99) (99)
Methanophagales archaeon isolate G37ANME1 NODE_1
39.42
(83)
48.46 42.00
(99) (84)
42.86 54.48 57.67 42.31
(94) (98) (99) (91)
37.02
(62)
No FwdF
32.28
(77)
52.67
(99)
37.17
(97)
55.62
(99)
55.68
(95)
Methanophagales archaeon isolate CONS3730B06UFb1
30.20
(89)
54.23 57.11
(96) (99)
39.02 53.94 56.06 43.82
(94) (99) (99) (91)
32.76
(62)
35.88
(98)
48.31 39.02 55.43
(94) (91) (98)
56.67
(99)
40.74
(83)
35.20
(66)
No FwdF
Candidatus Methanoperedens nitroreducens (ANME-2d)
57.09 45.66 65.11
(99) (100) (99)
58.33
(99)
47.58 51.84 53.18 44.79
(95) (99) (99) (95)
40.32
(79)
35.71
(53)
66.36 67.23
(99) (99)
42.67
(54)
35.53 33.33
(96) (92)
35.73
(98)

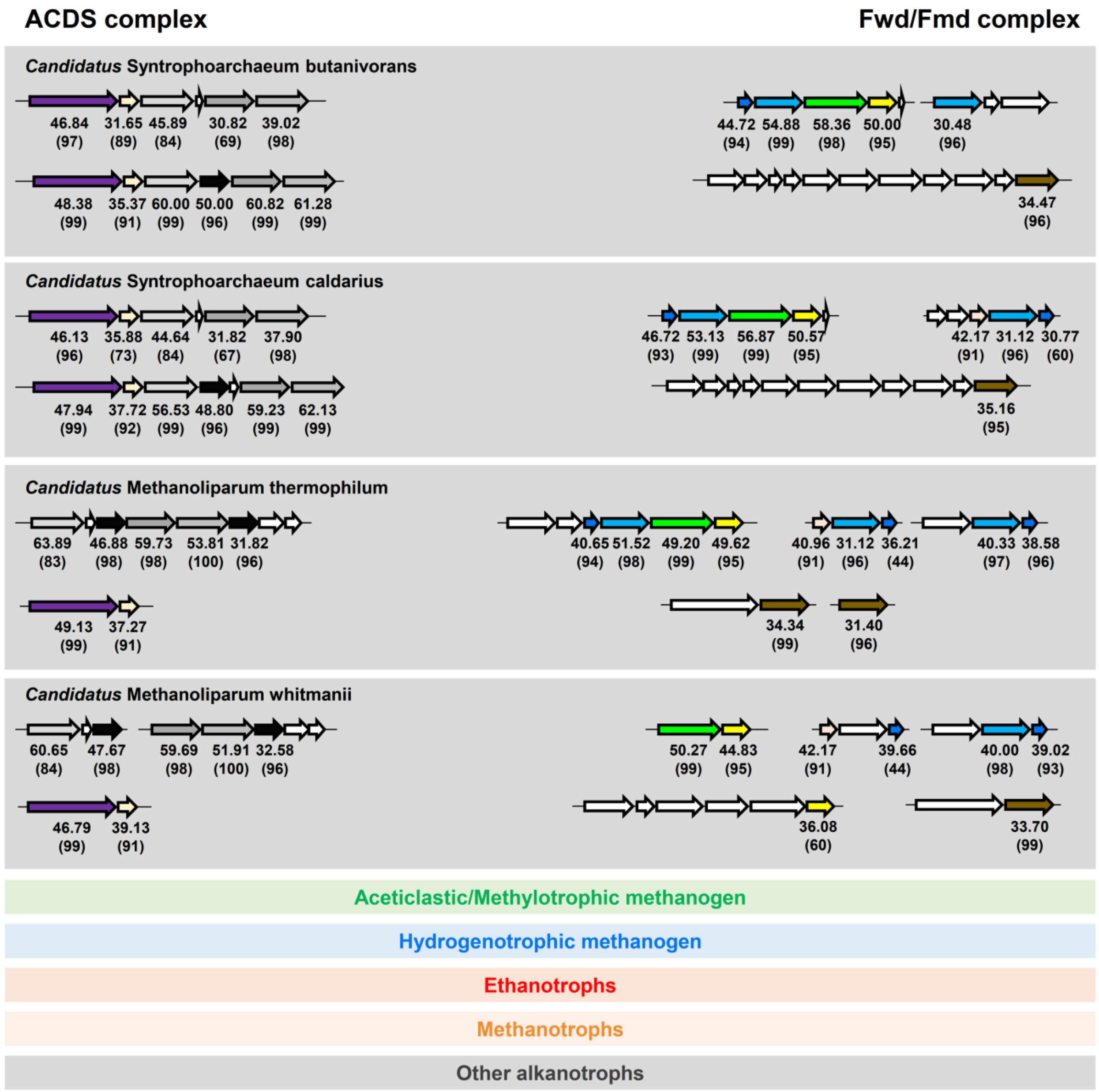


**Supplementary Fig. 16. Genomic environment of genes coding for ACDS and Fmd/Fwd subunits in methanogens, methanotrophs, ethanotrophs, and other alkanotrophs.** Genes are represented by arrows, and their size depends on the gene length. The genes coding for the α, ε, β, γ, δ, and ζ subunits of ACDS coloured deep purple, wheat, light grey, grey, dark grey, and orange, respectively, the maturation protein CooC in black, and the A, B, C, D, G, F and I subunits of the Fmd/Fwd complex in green, cyan, light yellow, marine blue, light pink, brown and dark orange, respectively. Other genes are coloured white. Gene annotation is based on sequence identity as determined by BLAST. The percentage of identity (and percentage of coverage) are given. Putative operon organisation, suggested by the Operon Mapper webserver[5], is represented by a continuous line. The background is coloured according to the main metabolism of the organism. The analysis suggests a putative $F_{420}$-reducing CODH in some ANME-1 species because of the presence of genes coding for a putative protein similar to the ζ subunit from the ethanotrophs.

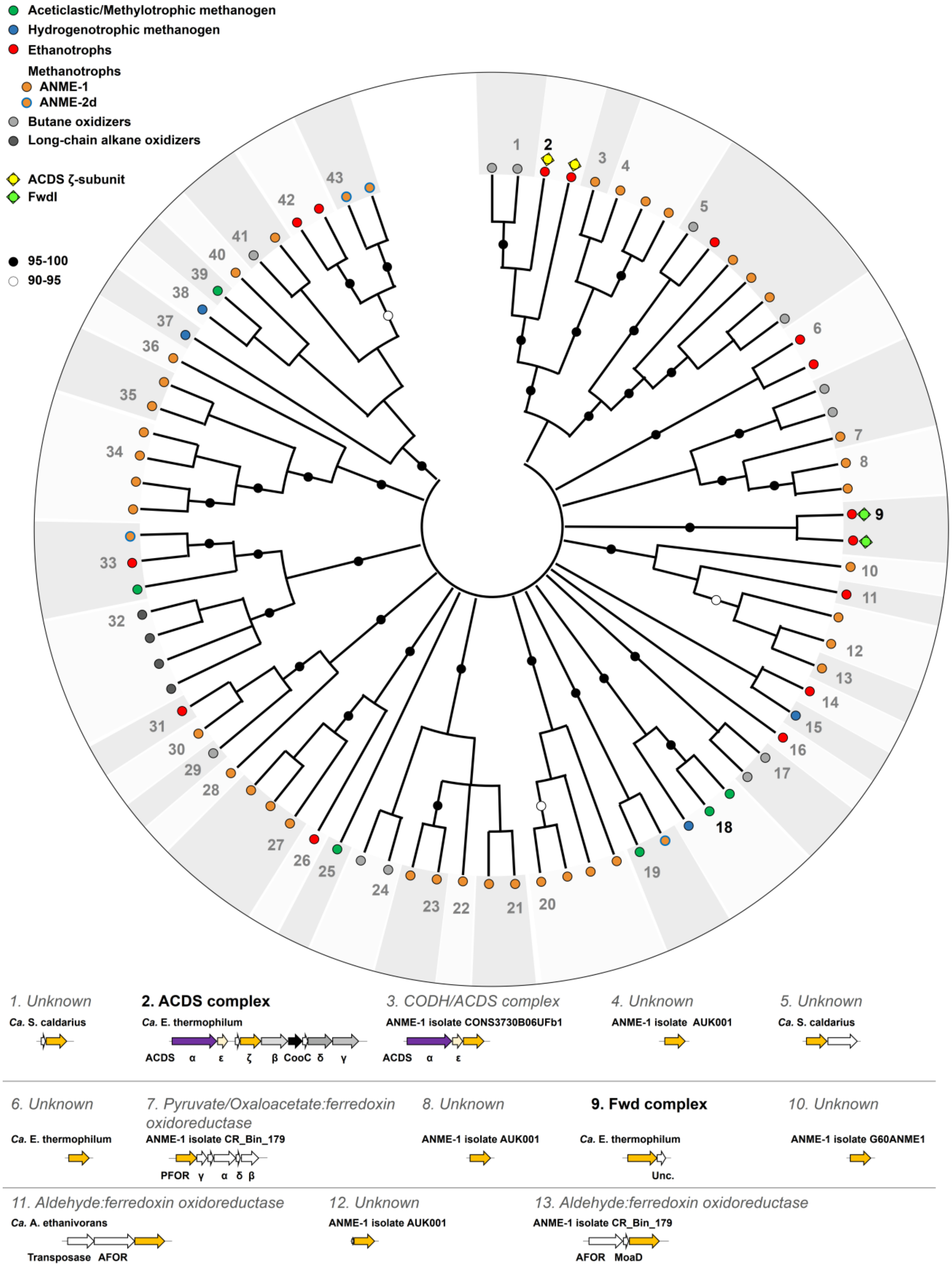

Aceticlastic/Methylotrophic methanogen
Hydrogenotrophic methanogen
Ethanotrophs
Methanotrophs
ANME-1
ANME-2d
Butane oxidizers
Long-chain alkane oxidizers
ACDS ζ-subunit
FwdI
95-100
90-95
1
2
3
4
5
6
7
8
9
10
11
12
13
14
15
16
17
18
19
20
21
22
23
24
25
26
27
28
29
30
31
32
33
34
35
36
37
38
39
40
41
42
43
1. Unknown
Ca. S. caldarius
2. ACDS complex
Ca. E. thermophilum
ACDS α ε ζ β CooC δ γ
3. CODH/ACDS complex
ANME-1 isolate CONS3730B06UFb1
ACDS α ε
4. Unknown
ANME-1 isolate AUK001
5. Unknown
Ca. S. caldarius
6. Unknown
Ca. E. thermophilum
7. Pyruvate/Oxaloacetate:ferredoxin oxidoreductase
ANME-1 isolate CR_Bin_179
PFOR γ α δ β
8. Unknown
ANME-1 isolate AUK001
9. Fwd complex
Ca. E. thermophilum
Unc.
10. Unknown
ANME-1 isolate G60ANME1
11. Aldehyde:ferredoxin oxidoreductase
Ca. A. ethanivorans
Transposase AFOR
12. Unknown
ANME-1 isolate AUK001
13. Aldehyde:ferredoxin oxidoreductase
ANME-1 isolate CR_Bin_179
AFOR MoaD

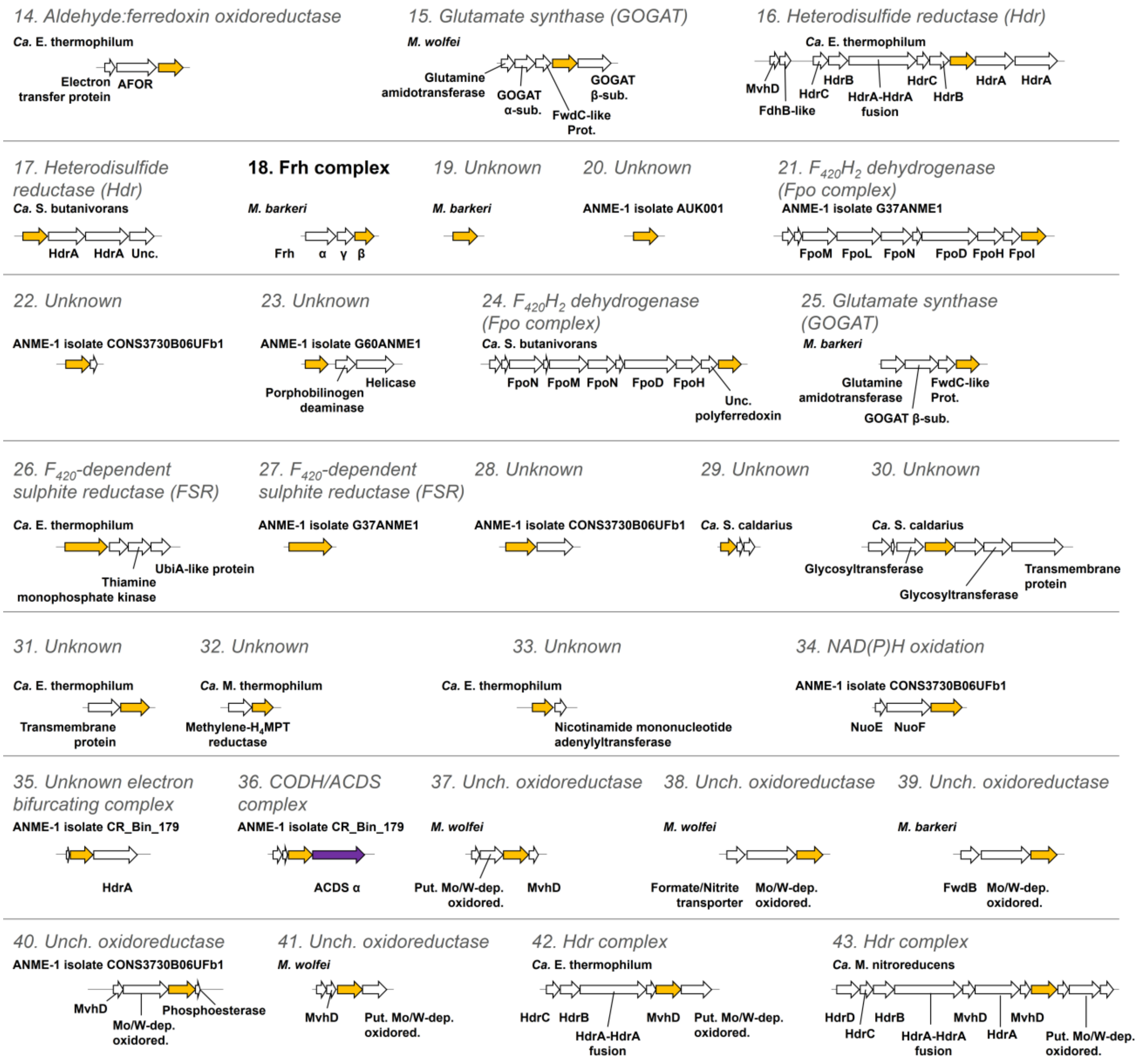


**Supplementary Fig. 17. Phylogenetic tree of $F_{420}$ reductase homologues and respective gene-environment in selected methanogens and alkanotrophs.** All sequences of $F_{420}H_2$ oxidases homologous to the ζ/FwdI subunits extracted from the genome of various organisms were used to construct a Maximum-likelihood evolution phylogenetic tree (top panel). Subgroups of the tree were established according to the genomic environment, shown in the bottom panel. Genes are represented by arrows with size depending on the gene length, and the gene coding for the α, ε, β, γ, and δ subunits of ACDS are coloured as in Fig. 2a. Gene annotation is based on sequence identity determined by BLAST using the SwissProt and PDB databases. Putative operon organisation, suggested by the Operon Mapper webserver [5], is represented by a continuous line. prot., sub., unc., put. and oxidored. stand for protein, subunit, uncharacterised, putative and oxidoreductase, respectively. The ζ subunit homologue encoded in the genome of *Ca.* Syntrophoarchaeum species (subgroup 1) may suggest an $F_{420}$-reducing ACDS for one of the two ACDS isoforms of the organisms (Supplementary Fig. 16). The second CODH isoform, which could include a ζ subunit found in other microorganisms, such as some ANME-1 species, might be involved in CO-detoxification, and further studies will have to clarify the roles of these proteins.

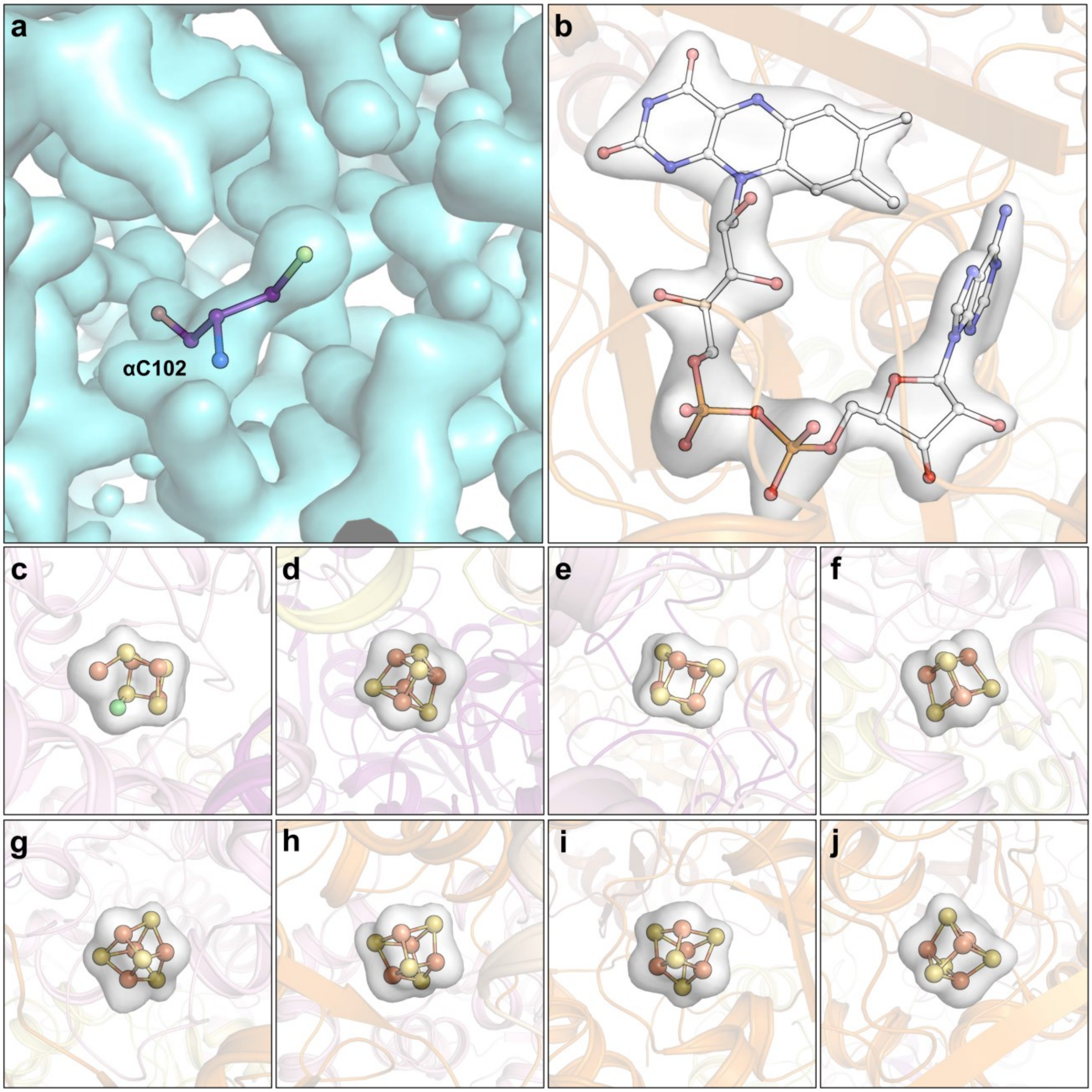


**Supplementary Fig. 18. Electron density and omit maps in the structure of the CODH component from *Ca*. E. thermophilum. a.** Electron density in the CODH component structure. The electronic density (contoured at 1 σ) around Cys102 in the α subunit is shown as a transparent cyan surface. Cys102 is shown as balls and sticks. This residue was selected as it is roughly located at the centre of the α subunit. **b-j.** Omit maps of the cofactors of the structures. The $F_o$–$F_c$ map (contoured at 5 σ) around FAD (**b**), C-cluster (**c**), cluster 1 (**d**), cluster 2 (**e**), cluster 3 (**f**), cluster 4 (**g**), cluster 5 (**h**), cluster 6 (**i**) and cluster 7 (**j**). This numbering is shown in Fig. 2 and Supplementary Fig. 6. The α, ε and ζ subunits are represented as transparent cartoons coloured purple, light yellow and orange, respectively. The different cofactors and Cys102 are represented in balls and sticks with carbon, nitrogen, oxygen, sulphur, phosphorus, nickel, and iron coloured white (purple for Cys102), blue, red, yellow, light orange, green, and orange, respectively.

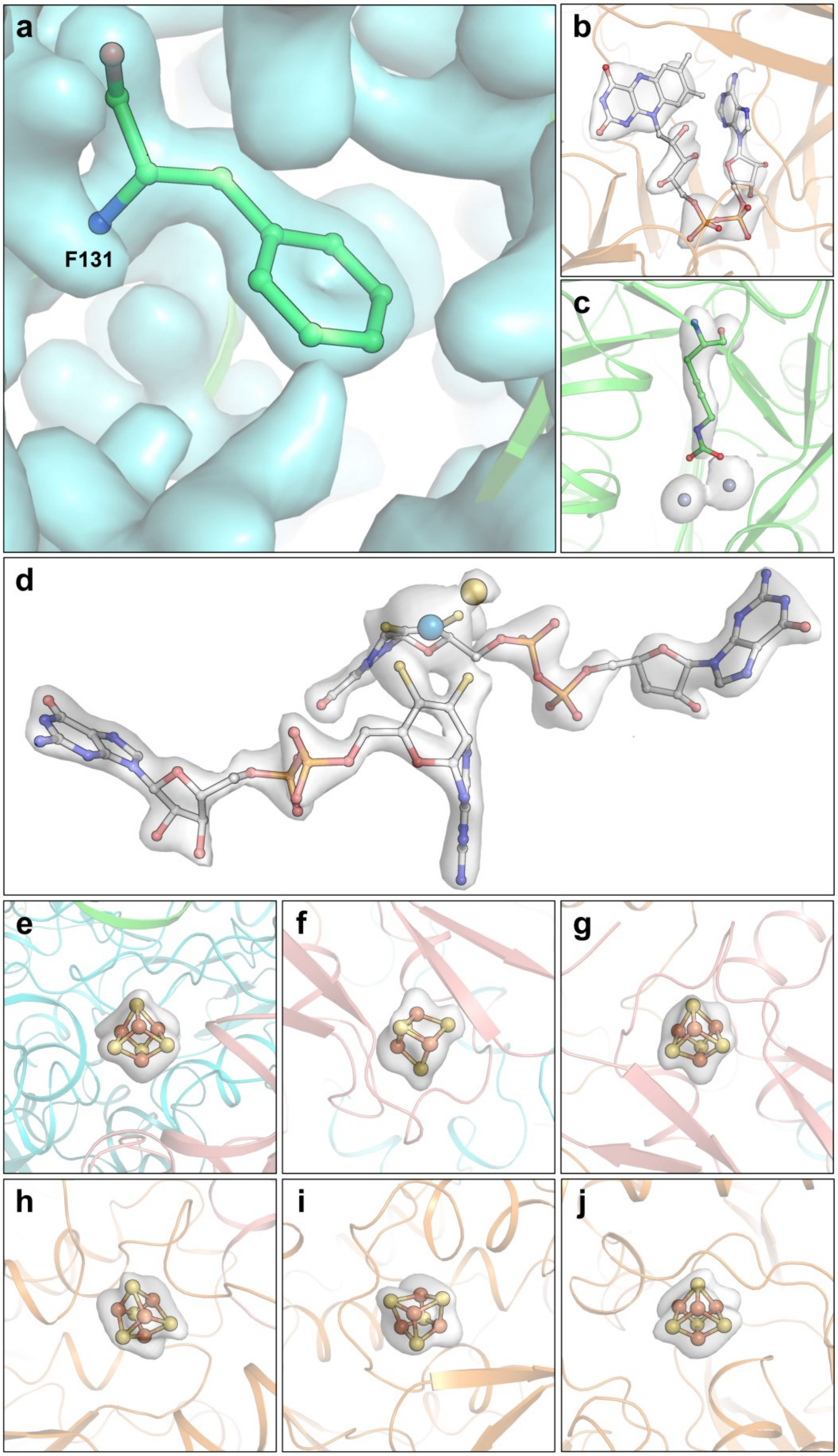
a
F131
b
c
d
e
f
g
h
i
j

**Supplementary Fig. 19. Electron density and omit maps in the Fwd structure from *Ca.* E. thermophilum. a.** Electron density in the Fwd structure. The electronic density (contoured at 1 σ) around Phe131 in the A subunit is shown as a transparent cyan surface. Phe131 is shown as balls and sticks. This residue was selected as it is roughly located at the centre of the A subunit. **b-j.** Omit maps of the cofactors of the structures. The $F_o$–$F_c$ map (contoured at 5 σ) around FAD (**b**), [Zn-Zn] binuclear site and the adjacent carboxylysine (**c**), Tungstopterin cofactor (**d**), cluster 1 (**e**), cluster 2 (**f**), cluster 3 (**g**), cluster 4 (**h**), cluster 5 (**i**) and cluster 6 (**j**). This numbering is shown in Fig. 3 and Supplementary Fig. 11. The A, B, C, D, G, and I subunits are represented as transparent cartoons coloured green, cyan, light yellow, marine blue, light pink, and orange, respectively. The different cofactors and Phe131 are represented in balls and sticks with carbon, nitrogen, oxygen, sulphur, phosphorus, zinc, tungsten and iron coloured white (green for Phe131), blue, red, yellow, light orange, grey, greyish blue and orange, respectively. For clarity, the proteins were not shown in panel d.

**Supplementary Table 1. Purification of the CODH component of the ACDS and Fwd of *Ca.* E. thermophilum.** Average and standard deviation are presented (n = 3 or 4 independent measurements, see Source Data file). An enzymatic unit refers to µmol of substrate oxidised per minute.

| Fraction, purification step | Total units (Units) | Protein (mg) | Specific activity (Units per mg of proteins) | Yield (%) | Purification (fold) |
|---|---|---|---|---|---|
| **CODH component of the ACDS** | | | | | |
| **Soluble extract** | 90.83 ± 7.40 | 105.42 | 0.86 ± 0.07 | 100 | 1.00 |
| **Q-Sepharose** | 56.05 ± 6.38 | 48.28 | 1.16 ± 0.13 | 61.71 | 1.35 |
| **Source 15PHE** | 21.92 ± 1.63 | 2.78 | 7.88 ± 0.59 | 24.13 | 9.15 |
| **Source 15PHE** | 19.73 ± 2.14 | 2.07 | 9.53 ± 1.03 | 21.72 | 11.07 |
| **Superdex 200 10/300** | 10.40 ± 1.81 | 0.82 | 12.69 ± 2.20 | 11.46 | 14.73 |
| | | | | | |
| **Fwd complex** | | | | | |
| **Furfurylformamide dehydrogenase activity** | | | | | |
| **Soluble extract** | 37.54 ± 7.61 | 105.42 | 0.36 ± 0.07 | 100 | 1.00 |
| **Q-Sepharose** | 5.90 ± 0.64 | 48.28 | 0.12 ± 0.01 | 15.73 | 0.34 |
| **Source 15PHE** | 2.47 ± 0.12 | 0.55 | 4.46 ± 0.21 | 6.57 | 12.52 |
| **Superdex 200 10/300** | 2.02 ± 0.11 | 0.40 | 5.05 ± 0.27 | 5.38 | 14.18 |
| **Formate dehydrogenase activity** | | | | | |
| **Soluble extract** | 130.27 ± 10.27 | 105.42 | 1.24 ± 0.10 | 100 | 1.00 |
| **Q-Sepharose** | 3.59 ± 0.33 | 48.28 | 0.07 ± 0.01 | 2.76 | 0.06 |
| **Source 15PHE** | 0.07 ± 0.00 | 0.55 | 0.13 ± 0.01 | 0.05 | 0.10 |
| **Superdex 200 10/300** | 0.05 ± 0.01 | 0.40 | 0.13 ± 0.03 | 0.04 | 0.11 |

**Supplementary Table 2. X-ray analysis statistics for the structures obtained from *Ca*. E. thermophilum.**

| | $\alpha_2\varepsilon_2\zeta_2$ ACDS subcomplex SAD Fe K-edge | $\alpha_2\varepsilon_2\zeta_2$ ACDS subcomplex | Fwd complex |
|---|---|---|---|
| Data collection | | | |
| Synchrotron source and beamline | SLS, X06DA | SOLEIL, PROXIMA-1 | SLS, X06DA |
| Wavelength (Å) | 1.73981 | 0.97856 | 1.00000 |
| Space group | $C222_1$ | $P2_12_12_1$ | $P2_1$ |
| Resolution (Å) | 57.27 – 3.00 (3.05 – 3.00) | 122.41 – 1.89 (2.10 – 1.89) | 57.38 – 1.97 (2.19 – 1.97) |
| Cell dimensions | | | |
| a, b, c (Å) | 109.40, 196.33, 494.09 | 97.07, 159.21, 191.44 | 107.63, 135.64, 149.90 |
| α, β, γ (°) | 90, 90, 90 | 90, 90, 90 | 90, 90.49, 90 |
| $R_{merge}$(%)[a] | 35.4 (171.5) | 14.9 (158.3) | 25.1 (120.0) |
| $R_{pim}$ (%)[a] | 9.9 (49.8) | 4.2 (46.0) | 10.1 (48.6) |
| $CC_{1/2}$[a] | 0.987 (0.590) | 0.999 (0.639) | 0.989 (0.621) |
| $I/\sigma_I$[a] | 7.3 (1.6) | 10.8 (1.7) | 7.2 (1.6) |
| Spherical completeness[a] | 100.0 (100.0) | 70.0 (13.0) | 55.7 (10.5) |
| Ellipsoidal completeness[a] | - | 95.9 (73.6) | 93.0 (64.3) |
| Redundancy[a] | 13.6 (12.7) | 13.6 (12.2) | 7.0 (6.9) |
| Nr. unique reflections[a] | 106,477 (5,263) | 164,775 (8,240) | 167,948 (8,397) |
| **Refinement** | | | |
| Resolution (Å) | - | 39.80 – 1.89 | 57.38 – 1.97 |
| Number of reflections | - | 164,728 | 167,913 |
| $R_{work}/R_{free}$[b] (%) | - | 16.51/18.73 | 17.57/21.00 |
| Number of atoms | | | |
| Protein (% completeness) | - | 20,153 (97.57) | 27,665 (99.39) |
| Solvent and ligands | - | 371 | 581 |
| Water | - | 1,418 | 2,282 |
| Mean B-value ($Å^2$) | - | 44.12 | 25.29 |
| Molprobity clash score, all atoms | - | 2.44 | 1.37 |
| Ramachandran plot | | | |
| Favoured regions (%) | - | 97.88 | 97.00 |
| Outlier regions (%) | - | 0.08 | 0.11 |
| r.m.s.d.[c] bond lengths (Å) | - | 0.009 | 0.010 |
| r.m.s.d.[c] bond angles (°) | - | 1.233 | 1.339 |
| PDB ID code | - | 8RIU | 8RJA |

[a] Values relative to the highest resolution shell are within parentheses. [b] $R_{free}$ was calculated as the $R_{work}$ for 5 % of the reflections that were not included in the refinement. [c] r.m.s.d., root mean square deviation.

**Supplementary Table 3. Peptide identification by Liquid chromatography MS-MS on the native electrophoresis gels.** The presented results correspond to the first hits, in order by intensity, after the removal of contaminants (e.g. keratin). iBAQ refers to intensity-based absolute quantification and is a measure of protein abundance.

| CODH component of the ACDS complex | | | | | |
|---|---|---|---|---|---|
| Accession | Protein | Peptide Number | Sequence coverage | Intensity | iBAQ |
| CAD7772032.1 | ACDS α subunit | 73 | 62.8 | 9.6982E+11 | 1E+10 |
| CAD7772047.1 | ACDS ζ subunit | 43 | 62.4 | 4.7723E+11 | 2E+10 |
| CAD7772037.1 | ACDS ε subunit | 18 | 81.6 | 2.8236E+11 | 1E+10 |

| Fwd complex | | | | | |
|---|---|---|---|---|---|
| Accession | Protein | Peptide Number | Sequence coverage | Intensity | iBAQ |
| CAD7768991.1 | FwdA | 50 | 83.2 | 3.1821E+11 | 8E+09 |
| CAD7768985.1 | FwdB | 37 | 69.1 | 2.8873E+11 | 1E+10 |
| CAD7775209.1 | FwdI | 24 | 53.2 | 2.2555E+11 | 1E+10 |
| CAD7768996.1 | FwdC | 24 | 90.1 | 8.1039E+10 | 4E+09 |
| CAD7768983.1 | FwdD | 8 | 44.4 | 3.0272E+10 | 5E+09 |

**Supplementary Table 4. Structural alignment of the CODH component of the ACDS and Fwd complex from *Ca.* E. thermophilum and related structures.** The alignment has been automatically performed with Pymol with default parameters, and the stretch of aligned sequences has been reported.

| Aligned structures (name, organism, PDB code, chains and residues) | Reference structure (name, organism, PDB code, chains and residues) | r.m.s.d. (Å) | Aligned Cα |
|---|---|---|---|
| α subunit ACDS, *M. barkeri*, 3CF4, A44-A803 | α ACDS subunit, *Ca.* E. thermophilum, 8RIU, A37-A787 | 0.720 | 681 |
| ε subunit ACDS, *M. barkeri*, 3CF4, B15-B167 | ε ACDS subunit, *Ca.* E. thermophilum, 8RIU, C12-C171 | 0.949 | 119 |
| αε ACDS, *M. barkeri*, 3CF4, A44-A804 and B15-B154 | αε subunits, *Ca.* E. thermophilum, 8RIU, A37-A788 and C12-C155 | 0.789 | 748 |
| | | | |
| FrhBG, *Methanothermobacter marburgensis*, 4OMF, A213-A273 and C2-C269 | ζ subunit, *Ca.* E. thermophilum, 8RIU, E16 -E74 and E84-E346 | 0.906 | 263 |
| Fsr, *Methanocaldococcus jannaschii*, 7NP8, A9-A291 | ζ subunit, *Ca.* E. thermophilum, 8RIU, E16-E306 | 1.264 | 211 |
| FwdI, *Ca.* E. thermophilum 8RJA, E7-E349 | ζ subunit, *Ca.* E. thermophilum, 8RIU, E7-E347 | 1.056 | 228 |
| FrhBG, *M. marburgensis*, 4OMF, A209-A271 and C6-C272 | FwdI, *Ca.* E. thermophilum, 8RJA, E6-E84 and E88-E351 | 1.311 | 251 |
| Fsr, *M. jannaschii*, 7NP8, A8-A326 | FwdI, *Ca.* E. thermophilum 8RJA, E11-E351 | 1.117 | 205 |
| | | | |
| FwdABCDG core, *M. wolfei*, 5T5M, A3-A566; B4-B429; C4-C261 and D1-D124 | FwdABCDG core, *Ca.* E. thermophilum, 8RJA, A3-A564; B4-B428; C4-C252 and J3-J124 | 0.791 | 1223 |
| FmdABCDG core, *M. hungatei*, 7BKB, I3-I568 and L10-L436 | FwdABCDG core, *Ca.* E. thermophilum, 8RJA, A3-A564 and B9-B426 | 0.991 | 829 |

**Supplementary Table 5. Expression level of genes proposed to be involved in ethanotrophy and putatively involved in $F_{420}$ reduction/$F_{420}H_2$ oxidation.** The transcriptomics data are extracted from Hahn *et al.*, 2020. The genes coding for the ACDS ζ subunit and FwdI are in bold.

| Locus tag | Name | Gene expression (RPKM) | | Rank |
|---|---|---|---|---|
| | | Average | Standard deviation | |
| Ethyl-Coenzyme M reductase (ECR) | | | | |
| FHEFKHOI_01410 | *mcrB* | 7,323.18 | 640.3673 | 18 |
| FHEFKHOI_01411 | *mcrG* | 6,676.097 | 845.1444 | 22 |
| FHEFKHOI_01412 | *mcrA* | 11,354.85 | 1371.936 | 9 |
| Acetyl-CoA decarbonylase/synthase (ACDS) | | | | |
| FHEFKHOI_01146 | *cdhA* | 2,182.283 | 33.01558 | 77 |
| FHEFKHOI_01147 | *cdhE* | 2,917.7 | 272.9681 | 52 |
| **FHEFKHOI_01149** | ***cdhZ*** | **2,785.547** | **170.5093** | **54** |
| FHEFKHOI_01150 | *cdhB* | 2,619.38 | 239.1818 | 58 |
| FHEFKHOI_01153 | *cdhD_1* | 4,056.627 | 193.9212 | 36 |
| FHEFKHOI_01154 | *cdhG* | 6,749.933 | 660.5352 | 21 |
| Methylenetetrahydromethanopterin reductase (Mer) | | | | |
| FHEFKHOI_00600 | *mer* | 3,442.87 | 323.0438 | 45 |
| Methylenetetrahydromethanopterin dehydrogenase (Mtd) | | | | |
| FHEFKHOI_01914 | *mtd* | 2,144.567 | 187.2971 | 79 |
| Methenyltetrahydromethanopterin cyclohydrolase (Mch) | | | | |
| FHEFKHOI_00609 | *mch* | 2,499.277 | 224.7675 | 64 |
| Formylmethanofuran-tetrahydromethanopterin formyltransferase (Ftr) | | | | |
| FHEFKHOI_02106 | *ftr* | 1,874.323 | 117.5848 | 94 |
| W-dependent Formylmethanofuran dehydrogenase (Fwd) | | | | |
| FHEFKHOI_00470 | *fwdD* | 1,584.31 | 187.9854 | 126 |
| FHEFKHOI_00471 | *fwdB* | 956.78 | 69.29146 | 223 |
| FHEFKHOI_00472 | *fwdA* | 1,297.407 | 60.08871 | 161 |
| FHEFKHOI_00473 | *fwdC* | 1,532.313 | 123.3891 | 131 |
| **FHEFKHOI_01727** | ***fwdI*** | **3,569.98** | **424.5134** | **41** |
| FHEFKHOI_00695 | *fwdG* | 920.5967 | 127.3583 | 236 |
| $F_{420}H_2$:quinone oxidoreductase (Fpo) | | | | |
| FHEFKHOI_00303 | *fpoA* | 1,591.543 | 221.9746 | 125 |
| FHEFKHOI_00304 | *fpoB* | 3,863.963 | 496.85 | 39 |
| FHEFKHOI_00305 | *fqoC* | 3,284.12 | 377.0664 | 47 |
| FHEFKHOI_00306 | *fqoD* | 1,486.367 | 103.957 | 138 |
| FHEFKHOI_00307 | *fpoH* | 1,684.537 | 52.04053 | 113 |
| FHEFKHOI_00308 | *fpoI* | 1,482.577 | 123.4509 | 139 |
| FHEFKHOI_00309 | *fpoJ* | 1,599.91 | 268.1379 | 124 |
| FHEFKHOI_00310 | CDS | 1,883.593 | 172.2733 | 93 |
| FHEFKHOI_00311 | *fpoK* | 2,104.413 | 179.1633 | 82 |
| FHEFKHOI_00312 | *fpoL_1* | 1,531.387 | 62.85304 | 132 |

| | | | | |
|---|---|---|---|---|
| FHEFKHOI_00313 | *fpoM_1* | 1,864.007 | 59.88921 | 97 |
| FHEFKHOI_00314 | *fpoN* | 2,646.033 | 68.66652 | 57 |
| FHEFKHOI_00315 | *fpoO* | 477.6033 | 87.51585 | 486 |
| Electron-confurcating heterodisulfide reductase (Hdr) | | | | |
| FHEFKHOI_01206 | *hdrA_2* | 1,252.033 | 35.98648 | 164 |
| FHEFKHOI_01207 | *hdrA_3* | 1,626.09 | 228.1921 | 117 |
| FHEFKHOI_01208 | *frhB* | 1,181.287 | 58.25349 | 181 |
| FHEFKHOI_01209 | *hdrB_2* | 1,281.423 | 96.00225 | 162 |
| FHEFKHOI_01210 | *hdrC_2* | 1,267.837 | 147.109 | 163 |
| FHEFKHOI_01211 | *hdrA_4* | 1,370.01 | 78.02363 | 152 |
| FHEFKHOI_01212 | *hdrB_3* | 853.58 | 70.98938 | 261 |
| FHEFKHOI_01213 | *hdrC_3* | 1,180.747 | 174.8528 | 182 |
| FHEFKHOI_01214 | *fdhB_1* | 1,767.580 | 155.71129 | 104 |
| FHEFKHOI_01215 | *mvhD* | 1,867.013 | 259.21779 | 95 |
| Aldehyde oxidoreductase (Aor) | | | | |
| FHEFKHOI_02073 | *dmsB* | 501.2867 | 85.31422 | 465 |
| FHEFKHOI_02074 | *aor_3* | 1,623.69 | 91.87875 | 118 |
| FHEFKHOI_02075 | *frhB* | 491.7167 | 76.1402 | 472 |
| Unknown | | | | |
| FHEFKHOI_01017 | *fpoF* | 1,449.643 | 159.1757 | 145 |
| FHEFKHOI_01018 | CDS | 651.22 | 87.53336 | 347 |
| Unknown | | | | |
| FHEFKHOI_01566 | CDS | 361.7333 | 45.57154 | 627 |
| FHEFKHOI_01567 | CDS | 292.99 | 34.71367 | 754 |
| FHEFKHOI_01568 | *frhB* | 181.48 | 19.39031 | 1029 |
| Unknown | | | | |
| FHEFKHOI_01073 | *frhB* | 299.22 | 21.00763 | 742 |
| Unknown | | | | |
| FHEFKHOI_00063 | *frhB (Fsr)* | 483.47 | 24.79062 | 482 |
| FHEFKHOI_00064 | *mqnD* | 415.7167 | 16.60141 | 549 |
| FHEFKHOI_00065 | *thiL_1* | 321.4333 | 34.62789 | 691 |
| FHEFKHOI_00066 | *ubiA_1* | 31.58 | 6.688101 | 1899 |
| Unknown | | | | |
| FHEFKHOI_01857 | CDS | 23.35667 | 4.415001 | 1967 |
| FHEFKHOI_01858 | *frhB* | 68.09333 | 6.015765 | 1623 |

## Supplementary References


1 Gong, W. *et al.* Structure of the $\alpha_2\varepsilon_2$ Ni-dependent CO dehydrogenase component of the *Methanosarcina barkeri* acetyl-CoA decarbonylase/synthase complex. *Proceedings of the National Academy of Sciences* **105**, 9558-9563, doi:10.1073/pnas.0800415105 (2008).

2 Lemaire, O. N. & Wagner, T. Gas channel rerouting in a primordial enzyme: Structural insights of the carbon-monoxide dehydrogenase/acetyl-CoA synthase complex from the acetogen *Clostridium autoethanogenum*. *Biochimica et Biophysica Acta Bioenergetics* **1862**, 148330, doi:10.1016/j.bbabio.2020.148330 (2021).

3 Doukov, T. I., Iverson, T. M., Seravalli, J., Ragsdale, S. W. & Drennan, C. L. A Ni-Fe-Cu Center in a Bifunctional Carbon Monoxide Dehydrogenase/ Acetyl-CoA Synthase. *Science* **298**, 567-572, doi:doi:10.1126/science.1075843 (2002).

4 Lemaire, O. N., Belhamri, M., Shevchenko, A. & Wagner, T. Carbon monoxide-driven bioethanol production operates via a tungsten-dependent catalyst. *bioRxiv*, doi:https://doi.org/10.1101/2024.07.29.605569 (2024).

5 Taboada, B., Estrada, K., Ciria, R. & Merino, E. Operon-mapper: a web server for precise operon identification in bacterial and archaeal genomes. *Bioinformatics* **34**, 4118-4120, doi:10.1093/bioinformatics/bty496 (2018).
