# Supplementary material for: Ethane-oxidising archaea couple CO2 generation to F420 reduction
Source: Nat Commun. 2024 Oct 21;15:9065. doi: 10.1038/s41467-024-53338-7 (PMC11493965; doi:10.1038/s41467-024-53338-7)
Supplement: Supplementary file 2 — Reporting Summary [file 41467_2024_53338_MOESM2_ESM.pdf]

nature portfolio

Corresponding author(s): Tristan Wagner

Last updated by author(s): Sep 25, 2024

# Reporting Summary

Nature Portfolio wishes to improve the reproducibility of the work that we publish. This form provides structure for consistency and transparency in reporting. For further information on Nature Portfolio policies, see our Editorial Policies and the Editorial Policy Checklist.

## Statistics

For all statistical analyses, confirm that the following items are present in the figure legend, table legend, main text, or Methods section.

| n/a | Confirmed | |
|---|---|---|
| ☐ | ☒ | The exact sample size (*n*) for each experimental group/condition, given as a discrete number and unit of measurement |
| ☐ | ☒ | A statement on whether measurements were taken from distinct samples or whether the same sample was measured repeatedly |
| ☒ | ☐ | The statistical test(s) used AND whether they are one- or two-sided<br>*Only common tests should be described solely by name; describe more complex techniques in the Methods section.* |
| ☒ | ☐ | A description of all covariates tested |
| ☒ | ☐ | A description of any assumptions or corrections, such as tests of normality and adjustment for multiple comparisons |
| ☐ | ☒ | A full description of the statistical parameters including central tendency (e.g. means) or other basic estimates (e.g. regression coefficient) AND variation (e.g. standard deviation) or associated estimates of uncertainty (e.g. confidence intervals) |
| ☒ | ☐ | For null hypothesis testing, the test statistic (e.g. *F*, *t*, *r*) with confidence intervals, effect sizes, degrees of freedom and *P* value noted<br>*Give P values as exact values whenever suitable.* |
| ☒ | ☐ | For Bayesian analysis, information on the choice of priors and Markov chain Monte Carlo settings |
| ☒ | ☐ | For hierarchical and complex designs, identification of the appropriate level for tests and full reporting of outcomes |
| ☒ | ☐ | Estimates of effect sizes (e.g. Cohen's *d*, Pearson's *r*), indicating how they were calculated |

*Our web collection on statistics for biologists contains articles on many of the points above.*

## Software and code

Policy information about availability of computer code

| | |
|---|---|
| Data collection | Spectrophotometer for activity measurement: Cary 60 UV-Vis Spectrophotometer Agilent technologies.<br>Plate reader: BMG Labtech FLUOstar Omega Microplate reader.<br>Synchrotron data collection are all stated in the Extended Data Table.<br>Mass spectrometry: Orbitrap Exploris 480 mass spectrometer (Thermo Fisher) |
| Data analysis | MEGA X version 10.1.8<br>autoPROC 1.0.5<br>CCP4 package 8.0.004<br>PHENIX 1.20.1_4487<br>COOT version 0.9.8.3 EL<br>Open-source Pymol Version 2.2.0<br>MaxQuant computational platform (version 2.0.10) |

For manuscripts utilizing custom algorithms or software that are central to the research but not yet described in published literature, software must be made available to editors and reviewers. We strongly encourage code deposition in a community repository (e.g. GitHub). See the Nature Portfolio guidelines for submitting code & software for further information.

## Data

Policy information about availability of data

All manuscripts must include a data availability statement. This statement should provide the following information, where applicable:

- Accession codes, unique identifiers, or web links for publicly available datasets
- A description of any restrictions on data availability
- For clinical datasets or third party data, please ensure that the statement adheres to our policy

All structures were validated and deposited in the Protein Data Bank (PDB) under the following accession numbers: 8RIU, Crystal structure of the F420-reducing carbon monoxide dehydrogenase component [https://www.rcsb.org/structure/8RIU] and 8RJA, Crystal structure of the F420-reducing formylmethanofuran dehydrogenase complex [https://www.rcsb.org/structure/8RJA]. All other data are available in the manuscript or the supplementary materials. Source data are provided in this work. The mass spectrometry raw data generated in this study have been deposited in the PRIDE database (EMBL-EBI) under accession code PXD054507 [https://www.ebi.ac.uk/pride/archive/projects/PXD054507]. The genomic sequences and the outputs of the Operon Mapper server generated in this study have been deposited in the Zenodo public database59 under accession code [https://zenodo.org/records/13381200].

## Research involving human participants, their data, or biological material

Policy information about studies with human participants or human data. See also policy information about sex, gender (identity/presentation), and sexual orientation and race, ethnicity and racism.

| | |
|---|---|
| Reporting on sex and gender | n.a. |
| Reporting on race, ethnicity, or other socially relevant groupings | n.a. |
| Population characteristics | n.a. |
| Recruitment | n.a. |
| Ethics oversight | n.a. |

Note that full information on the approval of the study protocol must also be provided in the manuscript.

# Field-specific reporting

Please select the one below that is the best fit for your research. If you are not sure, read the appropriate sections before making your selection.

☒ Life sciences ☐ Behavioural & social sciences ☐ Ecological, evolutionary & environmental sciences

For a reference copy of the document with all sections, see nature.com/documents/nr-reporting-summary-flat.pdf

# Life sciences study design

All studies must disclose on these points even when the disclosure is negative.

| | |
|---|---|
| Sample size | Sample size has been stated in the material and methods section.<br>For activity measurements, a relatively common number of replicates (n=3 to 4) was used at it allows to save the precious samples and to provided significant average and standard deviation values. Three independent protein purifications were carried out. |
| Data exclusions | Data were excluded only when there were evident experimental problems (e.g. oxygen contamination) or where controls indicated experimental flaws. |
| Replication | The activity measurements were performed at least in triplicates (n=3 to 4) from enzymes purified from a single purification process or extracts obtained from a single sample of frozen biomass. The presented data are representative of several distinct purification processes and different protein extractions (except for extracts from ANME species, whose cultivation time and yield did not allow to reproduce the experiment, as stated in the text). |
| Randomization | Randomization were applied to generate the Rfree set for the protein refinement. Except for structural analyses, randomization was not applied as it cannot be rationally applied on the sample preparation and measurements performed in this work. |
| Blinding | Blinding was not used in this study as the experiments are performed on samples freshly prepared in the laboratory. Even if the origin of samples was known, the experiments and data treatment were performed via standard and reproducible protocols to avoid bias. |

# Reporting for specific materials, systems and methods

We require information from authors about some types of materials, experimental systems and methods used in many studies. Here, indicate whether each material, system or method listed is relevant to your study. If you are not sure if a list item applies to your research, read the appropriate section before selecting a response.

## Materials & experimental systems

| n/a | Involved in the study |
|---|---|
| ☒ | ☐ Antibodies |
| ☒ | ☐ Eukaryotic cell lines |
| ☒ | ☐ Palaeontology and archaeology |
| ☒ | ☐ Animals and other organisms |
| ☒ | ☐ Clinical data |
| ☒ | ☐ Dual use research of concern |
| ☒ | ☐ Plants |

## Methods

| n/a | Involved in the study |
|---|---|
| ☒ | ☐ ChIP-seq |
| ☒ | ☐ Flow cytometry |
| ☒ | ☐ MRI-based neuroimaging |

# Plants

| | |
|---|---|
| Seed stocks | n.a. |
| Novel plant genotypes | n.a. |
| Authentication | n.a. |
